# New 4,5-Diarylimidazol-2-ylidene–iodidogold(I) Complexes with High Activity against Esophageal Adenocarcinoma Cells

**DOI:** 10.3390/ijms24065738

**Published:** 2023-03-17

**Authors:** Sebastian W. Schleser, Hindole Ghosh, Gerald Hörner, Jonathan Seib, Sangita Bhattacharyya, Birgit Weber, Rainer Schobert, Prasad Dandawate, Bernhard Biersack

**Affiliations:** 1 Organic Chemistry 1, University of Bayreuth, Universitätsstrasse 30, 95440 Bayreuth, Germany; 2 Cancer Biology, University of Kansas Medical Center, 3901 Rainbow Boulevard, Kansas City, KS 66160, USA; 3 Inorganic Chemistry IV, University of Bayreuth, Universitätsstrasse 30, 95440 Bayreuth, Germany

**Keywords:** gold, carbene ligands, metal-based drugs, anticancer agents, esophageal cancer

## Abstract

Inspired by the vascular-disrupting agent combretastatin A-4 and recently published anticancer active *N*-heterocyclic carbene (NHC) complexes of Au(I), a series of new iodidogold(I)–NHC complexes was synthesized and characterized. The iodidogold(I) complexes were synthesized by a route involving van Leusen imidazole formation and *N*-alkylation, followed by complexation with Ag_2_O, transmetalation with chloro(dimethylsulfide)gold(I) [Au(DMS)Cl], and anion exchange with KI. The target complexes were characterized by IR spectroscopy, ^1^H and ^13^C NMR spectroscopy, and mass spectrometry. The structure of **6c** was validated via single-crystal X-ray diffraction. A preliminary anticancer screening of the complexes using two esophageal adenocarcinoma cell lines showed promising nanomolar activities for certain iodidogold(I) complexes accompanied with apoptosis induction, as well as *c*-Myc and cyclin D1 suppression in esophageal adenocarcinoma cells treated with the most promising derivative **6b**.

## 1. Introduction

Esophageal cancer (EC) ranks seventh worldwide in terms of incidence (604,100 new cases) and sixth in overall mortality (544,076 deaths) [1]. Especially high morbidity and mortality rates for esophageal cancer are observed in East, Central, and South Asia, South and East Africa, and Northwest Europe. Moreover, these alarming numbers are expected to rise by 35–37% until 2030 [2]. Considering the poor prognosis of EC patients, frequently resulting from late diagnoses, this tumor is a considerable health issue for developed and developing countries [2,3]. EC is a rapidly growing cancer with a poor five-year survival rate of <20% [4]. The biology of EC is hardly understood compared to other cancers and typically shows extremely aggressive clinical features upon diagnosis [5]. Hence, it is one of the most challenging cancers to treat [6]. EC is divided into two histological subtypes [squamous cell carcinoma (ESCC) and adenocarcinoma (EAC)], which have diverse etiologies. ESCC is the most common EC worldwide, while EAC accounts for roughly two-thirds of EC cases in western countries [7]. The incidence of EAC is rapidly increasing across high-income countries due to obesity, an increase in gastroesophageal reflux disease (GERD), and Barrett’s esophagus (BE) [8]. Current treatment options for esophageal cancer include endoscopic and surgical treatments and chemoradiotherapy based on platinum complexes such as cisplatin [9,10]. Recently, the combination of platinum-based chemo(radio)therapy with immune checkpoint inhibitors revealed promising results in patients suffering from EC [11]. Thus, platinum complexes continue to be highly relevant for the management of EC, and metal-based drugs with other metals than platinum may have great potential for the treatment of this disease in the future.

**Figure 1:**
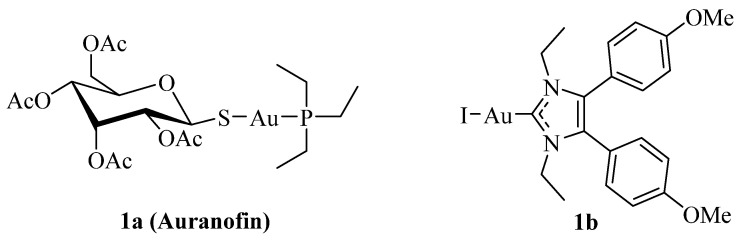
Structures of auranofin (**1a**) and the NHC–gold(I) complex **1b**.

While platinum complexes are clinically applied for the treatment of various solid tumors, gold complexes such as auranofin (**1a**) are being used for the treatment of rheumatoid arthritis (“chrysotherapy”) (Figure 1) [12]. However, as early as 1986, auranofin was investigated as an active compound in a study with esophageal carcinoma [13]. Auranofin revealed anticancer properties in preclinical studies based on its considerable inhibition of thioredoxin reductase (TrxR) associated with oxidative stress by the formation of reactive oxygen species (ROS), leading to increased apoptosis induction [12]. In addition, auranofin was described as an inhibitor of proteasomal deubiquitinase in cancer cells [13,14]. The mitochondrial Mia40/CHCHD4 pathway playing a crucial role in the oxidation of freshly imported cysteine-rich proteins in mitochondria was identified as a target of auranofin in fungi, which might be relevant in vigorously proliferating cancer cells too [15,16]. Furthermore, derivatives of auranofin with improved anticancer properties were described [17,18]. Recent developments in the field of auranofin-derived gold compounds led to highly active thiolatopurine complexes showing strong DNA damaging effects aside from TrxR inhibition, apoptosis induction, ROS formation, and antiangiogenic effects [19]. Advances in the research of *N*-heterocyclic carbene (NHC) gold complexes established a prospering class of metal-based drugs, which showed eminent anticancer activities based on TrxR inhibition and DNA interaction, adding well to the currently available arsenal of anticancer platinum complexes and the leading gold(I) complex auranofin [20,21,22,23,24]. Another emphasis was laid on the investigation of the effects on cytoskeletal structures, especially actin filaments and microtubules, by gold(I)–NHC complexes with 4,5-diarylimidazole-2-ylidene ligands derived from the tubulin-binding natural product combretastatin A-4 [25,26,27]. Meanwhile, the disruption of the actin cytoskeleton was described for cancer cells treated with auranofin too [28]. Recently, the relevance of the iodido ligand of neutral 4,5-dianisylimidazole-2-ylidene–iodidogold(I) complexes was proved, which revealed promising in vitro and in vivo antihepatoma activities of the iodido complex **1b** when compared with analogous gold(I)–NHC complexes bearing halo ligands (bromido), pseudohalo ligands (isocyanato), or acetato ligands (Figure 1) [29].

With iodido being the optimal ligand for neutral 4,5-dianisylimidazol-2-ylidene–gold(I) complexes in hepatoma, subtle changes in the 4,5-dianisylimidazole-based NHC ligand might lead to iodidogold(I) complexes with improved activities against various tumor entities. This work studied the impact of various alterations of the NHC ligand of **1b** on the activity against invasive EAC cells.

## 2. Results

### 2.1. Chemistry

**Scheme 1:**
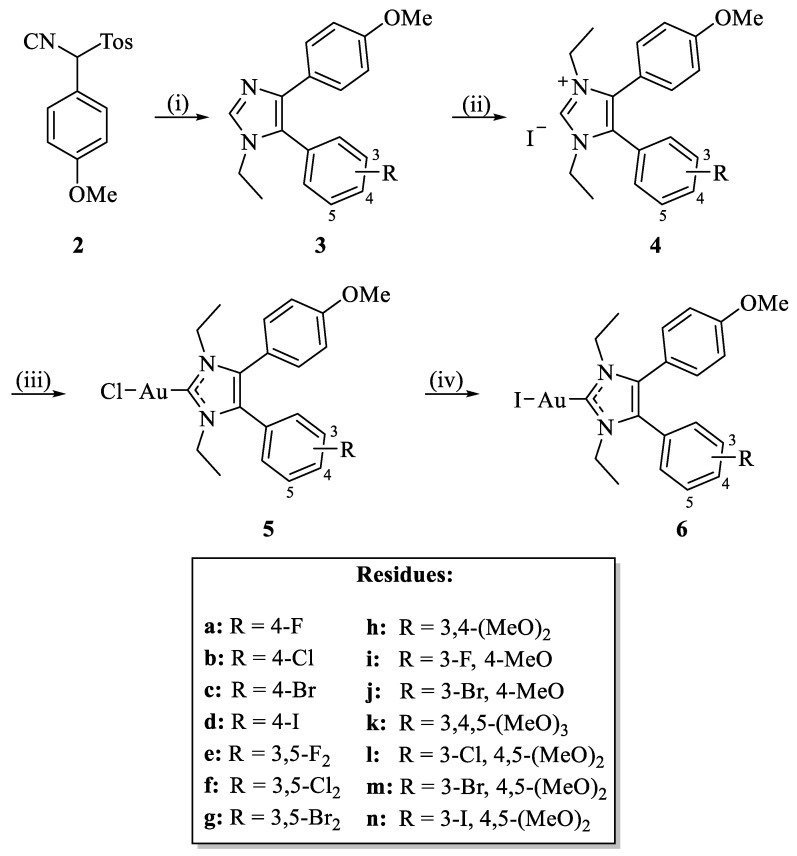
Reagents and conditions: (i) aryl aldehyde, 2M EtNH_2_/THF, AcOH, K_2_CO_3_, EtOH/DME, reflux, 7 h; (ii) EtI, MeCN, reflux, 24 h; (iii) (a) Ag_2_O, CH_2_Cl_2_, r.t., 24 h, (b) [Au(DMS)Cl], CH_2_Cl_2_, r.t., 24 h; (iv) KI, acetone, r.t., 24 h.

The synthesis of the new iodidogold(I) complexes **6a–n** was carried out following procedures from the literature (Scheme 1) [25,26,27,29]. The TosMIC reagent **2** was prepared as described before [30]. Reagent **2** was treated with EtNH_2_ and the corresponding aryl aldehyde to form the 1-ethyl-4,5-diarylimidazoles **3**. *N*-Alkylation with ethyl iodide led to the imidazolium iodides **4**. The reaction of **4** with Ag_2_O followed by transmetalation of the NHC–silver(I) intermediates with chloro(dimethylsulfide)gold(I) [Au(DMS)Cl] resulted in the chloridogold(I) complexes **5**. Finally, the chlorido ligands of **5** were replaced by iodido ligands upon reaction with KI, thus, generating the target complexes **6a–n**.

**Scheme 2:**
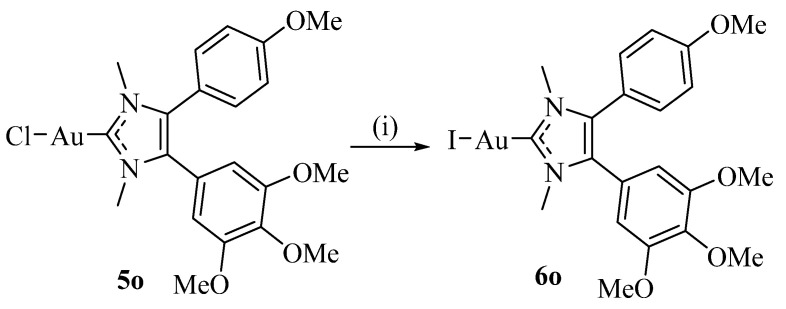
Reagents and conditions: (i) KI, acetone, r.t., 24 h.

In addition to the diethylimidazol-2-ylidene complexes **1b** and **6a–o**, the new **1b**-analogous dimethylimidazol-2-ylidene complex **6o** was prepared from the known chloridogold(I) precursor **5o** for comparison purposes (Scheme 2) [24].

**Figure 2:**
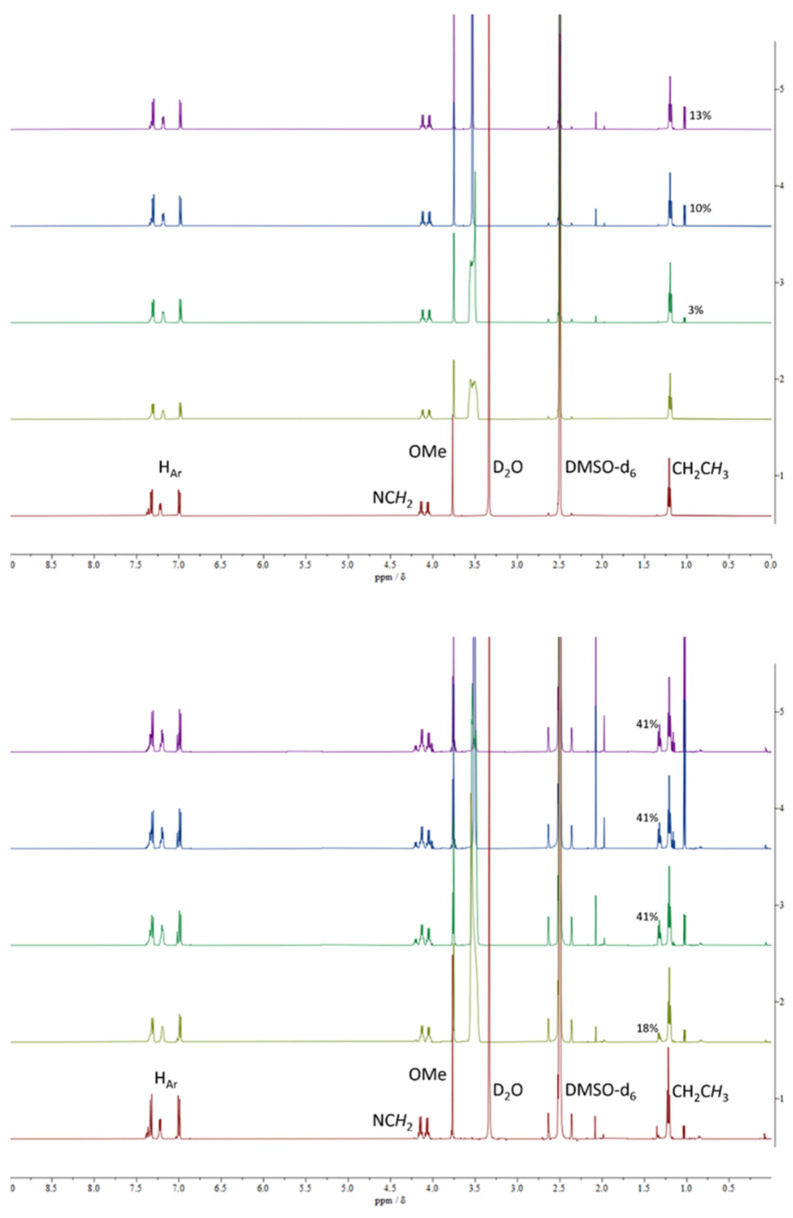
^1^H-NMR spectra of **5e** (top) and **6e** (bottom) in DMSO-d_6_ after 0 h (red), +5% D_2_O 0 h (yellow), 24 h (green), 48 h (blue), and 72 h (purple) with assignment. The hydrolysis product is given as a percentage averaged from the integrals of the additional peaks. These were set in a 1:1 ratio to the product peaks, assuming the same number of protons and approximately the same molar mass.

The stability of complexes **5e** and **6e** was studied by ^1^H NMR spectroscopy in DMSO-d_6_ upon addition of 5% D_2_O (Figure 2). After 24 h, new signals of the *N*-ethyl groups appeared, which grew with prolonged incubation. These new signals can be assigned to DMSO coordination and hydrolysis products of the iodidogold(I) complexes. However, the gold complexes possess considerable stability in aqueous solvents even after longer incubation since only a tiny fraction of complexes underwent hydrolysis by that time.

**Figure 3:**
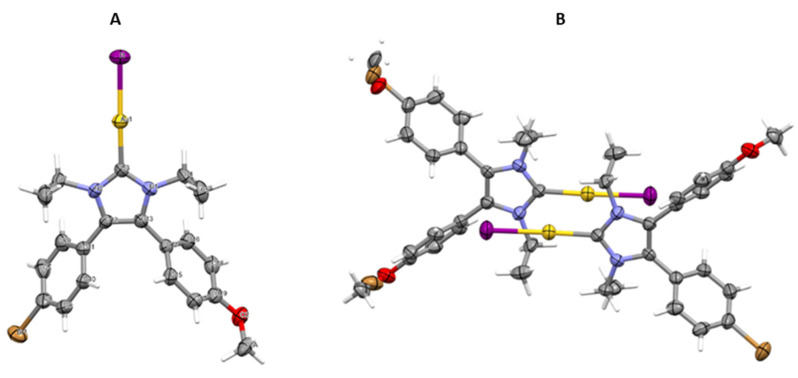
(**A**) Crystal structure of the iodidogold(I) complex **6c**. Selected bond lengths (Å) and angles (°): Au1–I1, 2.5567(6); Au1–C3, 2.002(7); N1–C3, 1.351(8); N2–C3, 1.342(8); C3–Au1–I1, 174.30(18); N2–C3–Au1, 128.4(5); N1–C3–Au1, 126.4(4); N2–C3–N1, 104.9(6); (**B**) pseudo-dimer of **6c** viewed along crystallographic axis *a* (ellipsoids plotted at 50% probability); positional disorder in one of the complex modules causes an apparent overlap of methoxy and bromo substituents; crystallographic data of the structure have been deposited at the Cambridge Crystallographic Data Centre: CCDC-2235874.

Single crystals suitable for X-ray diffraction structure elucidation were obtained via slow diffusion of *n*-hexane into a saturated solution of **6c** in CHCl_3_ at 4 °C. The **6c** crystallizes in the monoclinic space group *P2*_1_/*n* with Z = 4. A plot of the molecular structure of **6c** is given in Figure 3A. The linearity of the I–Au–C vector and the bond distance between Au and the carbene carbon are well in agreement with values from the literature [31]. Selected bond lengths and angles are given in the caption of Figure 3; crystallographic details are assembled in Table 1. A view along crystallographic axis *a* reveals the formation of pseudo-dimers in a head-to-tail arrangement with a short Au–Au non-bonded contact of *d*(Au–Au) = 3.806 Å (Figure 3B).

### 2.2. Activity against Esophageal Cancer

**Figure 4:**
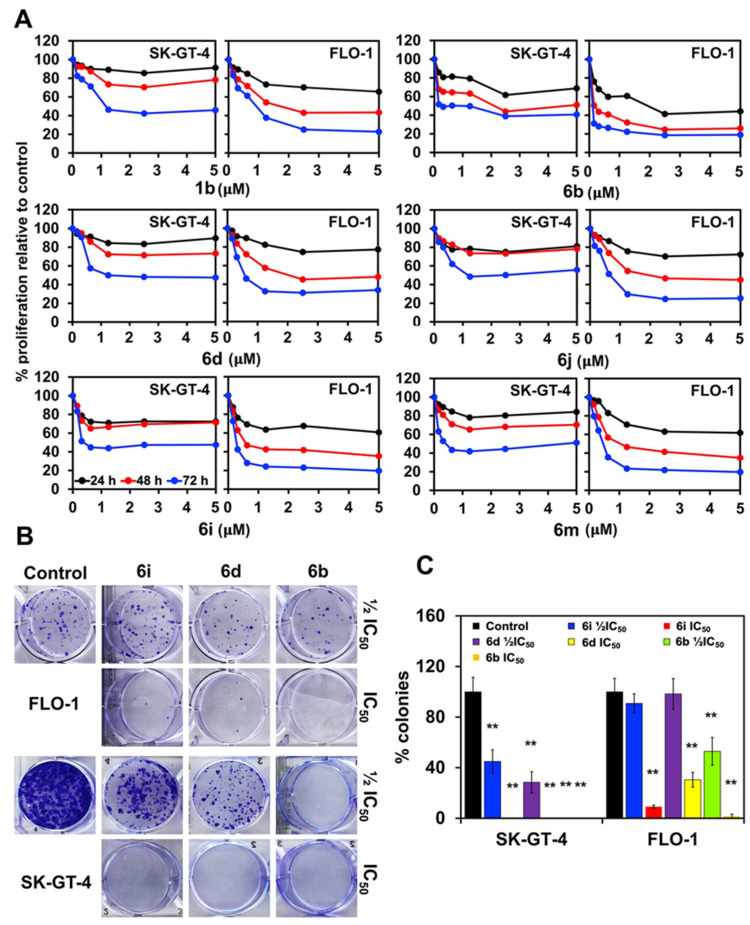
Gold complexes **6b**, **6d**, and **6i** inhibited the proliferation and colony formation in EAC cell lines. (**A**) Gold complexes inhibited the proliferation of SK-GT-4 and FLO-1 cells in a time- and dose-dependent manner. Gold complexes (semi-IC_50_ and IC_50_ concentrations) inhibited colony formation both in size (**B**) and number (**C**) in EAC cell lines. ** *p* < 0.01.

Initially, the antiproliferative activities of complexes **6a–o** against cell lines (FLO-1 and SK-GT-4) were evaluated using the hexosaminidase assay (Table 2 and Figure 4A). The known iodido complex **1b** and the chlorido complexes **5a–g** were analyzed for comparison. Except for complex **6l**, all iodidogold(I) complexes **6** exhibited moderate to high activities against FLO-1 cells, while the SK-GT-4 cells were less sensitive in most cases. Complexes **6b**, **6i**, and **6m** were the most active compounds that inhibited the growth of EAC cells in a dose- and time-dependent manner, with IC_50_ values in the nanomolar concentration range of 0.26–0.4 µM and 0.12–0.45 µM in the cases of SK-GT-4 and FLO-1, respectively. These complexes were also distinctly more active than the known complex **1b**. In analogy to the high activity of the 4-chlorophenyl derivative **6b**, which was the most active compound of this series, the 3,5-dichlorophenyl derivative **6f** was distinctly more active against the SK-GT-4 cells than its close congeners **6e** and **6g**. In contrast, the 3-chloro-4,5-dimethoxyphenyl analog **6l** was inactive. The chloridogold(I) complexes **5** were generally less active than their iodidogold(I) analogs, except for complexes **5a** and **5g**, which were more active against SK-GT-4 cells than the iodido complexes **6a** and **6g**. In addition, complex **6o**, the *N*,*N*-dimethyl analog of **6k**, was more active than **6k**.

Compounds **6b**, **6d**, and **6i** were selected for further evaluation of their inhibitory activity against EAC cell lines (SK-GT-4 and FLO-1). The colony formation was performed to understand the long-term effect of these gold complexes on EAC cells. Complex **6b** showed the strongest colony formation suppression (i.e., reduced colony size and number) at IC_50_ and semi-IC_50_ concentrations. It completely inhibited colony formation in terms of size and number (*p* < 0.01) at its IC_50_ dose after treatment for 72 h (Figure 4B,C). In addition, at IC_50_ doses, the colony formation was almost completely inhibited by complex **6i**, while **6d** was less inhibitory. These data suggested that the anticancer effects of the tested gold complexes are irreversible.

**Figure 5:**
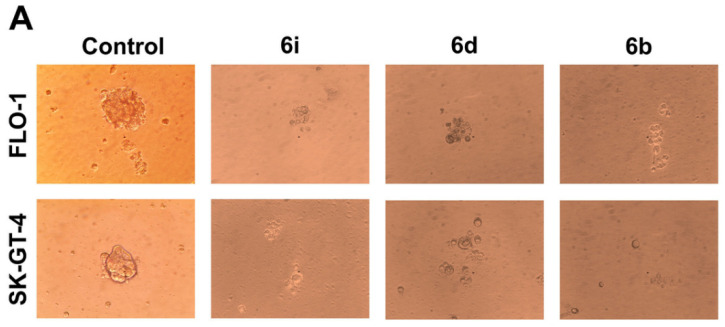

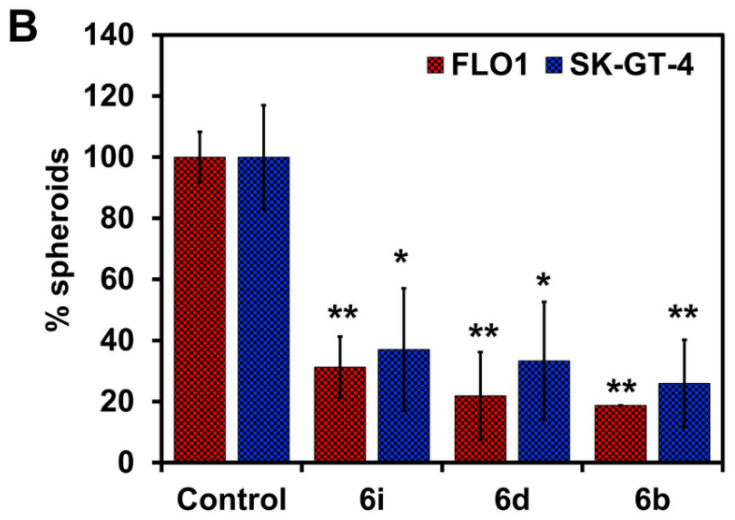
Inhibition of EAC cell spheroid formation by test compounds **6b**, **6d**, and **6i** (IC_50_ concentrations). The gold complexes reduced (**A**) size and (**B**) number of spheroids. The spheroids were photographed at 10× magnification. * *p* < 0.05, ** *p* < 0.01.

**Figure 6:**
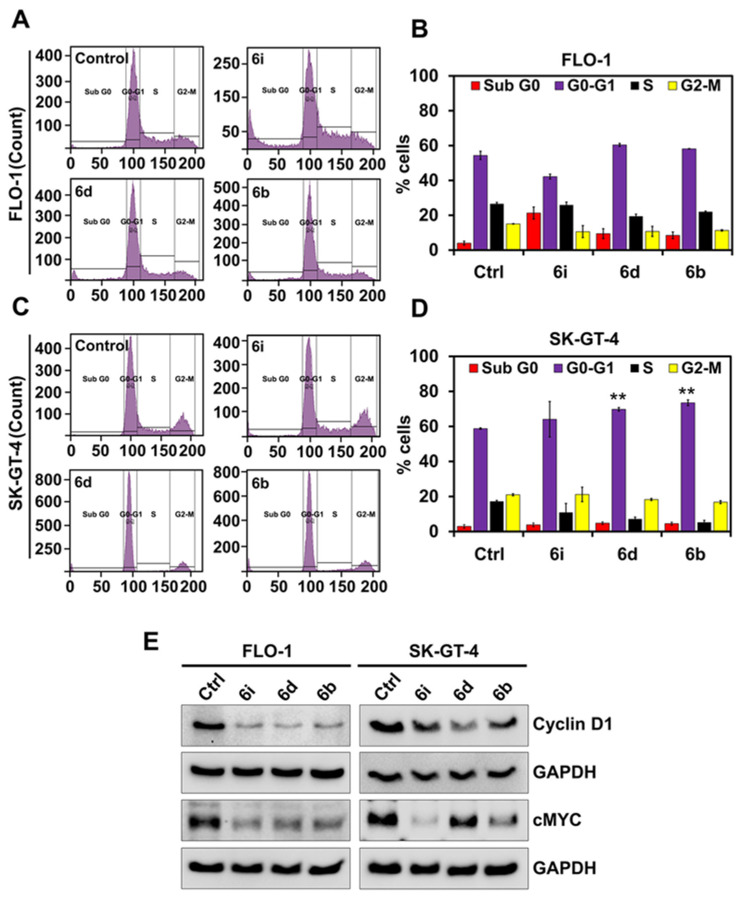
Gold complexes induced cell cycle arrest in EAC cells. (**A**–**D**) Gold complexes **6b**, **6d**, and **6i** (IC_50_ concentrations) induced G0–G1 cell cycle arrest in SK-GT-4 cells. (**E**) Gold complexes **6b**, **6d**, and **6i** (IC_50_ concentrations) inhibited cyclin D1 and c-Myc expression in SK-GT-4 and FLO-1 cells. ** *p* < 0.01.

**Figure 7:**
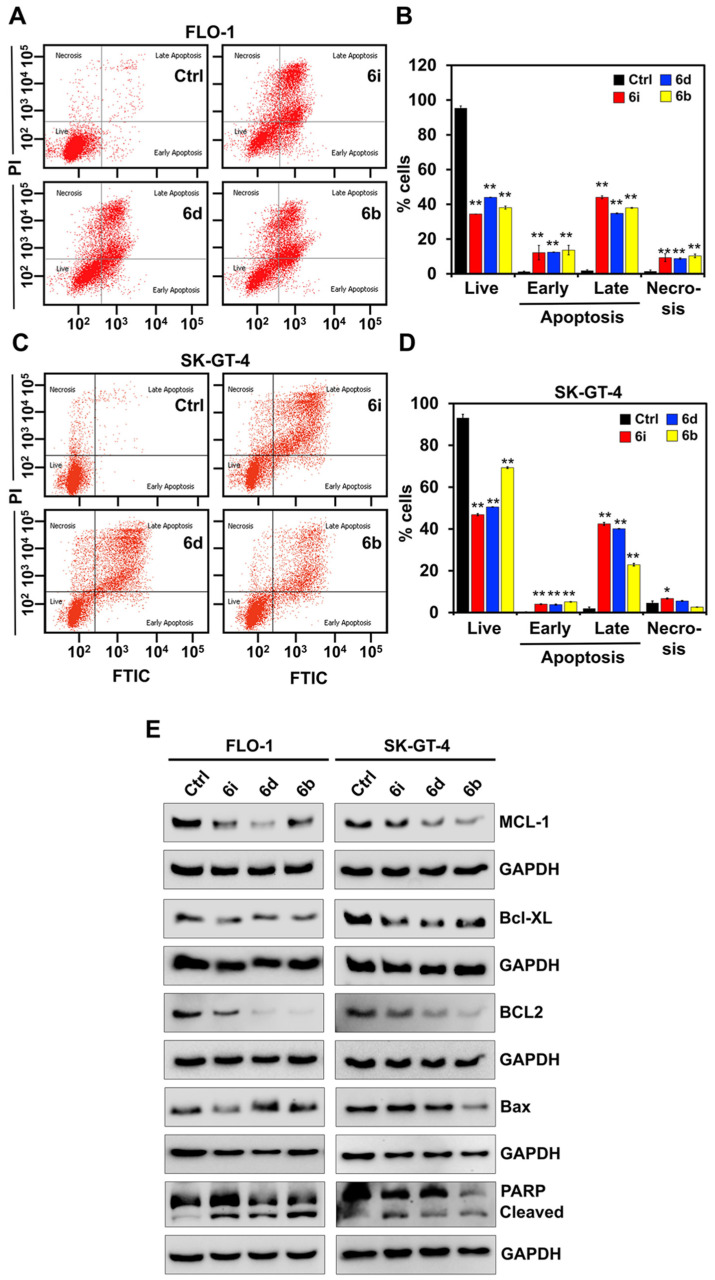
Gold complexes induced apoptosis in EAC cells. (**A**–**D**) Gold complexes **6b**, **6d**, and **6i** (IC_50_ concentrations) induced late-phase apoptosis in SK-GT-4 and FLO-1 cells, as assessed by an Annexin-PI assay using flow cytometry. (**E**) Cell lysates from EAC cells, when treated with complexes **6b**, **6d**, and **6i** (IC_50_ concentrations), showed significant cleavage of PARP compared to untreated controls. The treatment also reduced anti-apoptotic markers Bcl-XL, Mcl-1, and Bcl-2. * *p* < 0.05, ** *p* < 0.01.

Several studies showed cancer stem cells (CSCs) are involved in tumor initiation, aggressiveness, and drug resistance in EAC. Hence, targeting CSCs is an attractive strategy to treat [32]. It was observed that CSCs form spheroids in ultra-low attachment plates. Hence, we used a spheroid formation assay to assess the effects of gold complexes on EAC CSCs. The complexes **6b**, **6d**, and **6i** inhibited spheroid formation (both size and numbers) by EAC cells (Figure 5A,B). Complexes **6b** and **6d** showed the strongest inhibitory effects on spheroids. Next, the highly antiproliferative complexes **6b**, **6d**, and **6i** (IC_50_ concentration, 72 h time-point) were selected and tested for their effects on the cell cycle of EAC cells using flow cytometry (Figure 6A–D). All three compounds induced cell cycle arrest in SK-GT-4 and FLO-1 cells. Specifically, treatment with compounds **6b** and **6d** induced G0–G1 cell cycle arrest in SK-GT-4 cells (*p* < 0.01), while compound **6i** led to accumulation of FLO-1 cells in the sub-G0 phase of the cell cycle. The accumulation of cells in the sub-G0 phase after gold complex treatment can be the consequence of fragmented DNA, indicating the cytotoxic effects on EAC cell lines. To understand the mechanistic changes in proteins due to cell cycle arrest, we performed a Western blot to study the levels of cyclin D1 and cMyc. Cyclin D1 is known to drive cell cycle progression, while *c*-Myc regulates cyclin D1 to induce proliferation and tumor growth [33]. Moreover, these complexes suppressed the expression of *c*-Myc and cyclin D1, which is in line with their strong cell death/apoptosis induction (Figure 6E). Hence, we further studied the ability of **6b**, **6d**, and **6i** (IC_50_ concentration) to induce apoptosis in EAC cell lines using the Annexin V/PI assay and flow cytometry. Cell populations treated with the gold complexes (IC_50_ concentration) for 72 h showed increased percentages of apoptotic (especially late apoptotic) and necrotic cells when compared with untreated control populations (Figure 7A–D, *p* < 0.01). In line with this finding, the number of viable cells was reduced in the treated populations. The Western blot analysis showed that all three gold(I) complexes (IC_50_ concentration) increased cleaved PARP as a sign of apoptosis after 72 h. The expression of the anti-apoptotic factors Bcl-XL, Bcl-2, and Mcl-1 was suppressed in cells treated with the gold complexes. No significant differences in pro-apoptotic Bax expression were observed compared to untreated cells (Figure 7E). These data suggest that gold complexes induced apoptosis by inhibiting the apoptotic proteins involved in cancer cell survival and, hence, can be used in the combination with current chemotherapeutic agents.

## 3. Discussion

New derivatives of the published 4,5-dianisylimidazol-2-ylidene–iodidogold(I) complex **1b** were prepared by changing the modification of one of the ligand’s anisyl residues. The synthesis of the new iodidogold(I)–NHC complexes **6a–o** was straightforward and based on previously published works by our groups and by Bian and coworkers [25,26,27,29]. In this way, many highly antitumoral iodidogold(I)–NHC complexes were identified. The antiproliferative activities of several new iodidogold(I) complexes against two invasive EAC cell lines were superior to the activity of the known complex **1b**. They were also much more active than their chloridogold(I) precursors. Neutral chloridogold(I)–NHC complexes were often reported to be less anticancer active than analogous cationic NHC–gold(I) complexes [34,35]. Chloridogold–NHC complexes were casually developed as selective antiparasitic agents due to their relatively low toxicity to human cells [36]. However, with the exchange of the chlorido ligand for an iodido ligand, the cytotoxicity of neutral iodidogold(I) complexes reached excellent IC_50_ values in cancer cells, being in the active concentration range of known cationic triphenylphosphinogold(I)–NHC complexes and biscarbene–gold(I) complexes. Future studies will reveal how far neutral iodidogold(I)–NHC complexes and cationic NHC–gold(I) complexes differ in their performance in cancers in terms of activity, cellular localization/accumulation, and mechanisms of action. Nevertheless, it is noteworthy that, already, slight modifications of one of the phenyl rings of the 4-anisyl-5-arylimidazole-based NHC ligand system applied in this study can lead to strong changes in the anticancer activity of the tested iodidogold(I) complexes. For instance, while the 4-bromophenyl derivative **6c** was virtually inactive against the SK-GT-4 cells, its close 4-chlorophenyl analog **6b** is the most active complex identified in this study. In addition, while complexes **1b**, **6b**, **6d**, **6i**, and **6m** showed high antiproliferative activities against FLO-1 cells and SK-GT-4 cells, the FLO-1 cells were much more sensitive to certain complexes such as **6a**, **6c**, **6e** and **6g** than the SK-GT-4 cells. FLO-1 cells are p53-mutant cells, and the gold complexes may take advantage of the absence of p53, the “guardian of the genome”, to kill the FLO-1 cells. Analogously, higher sensitivities of p53-knockout HCT-116 colon carcinoma cells, when compared with p53-wildtype HCT-116 cells, were observed only recently for various NHC–gold(I) complexes and phosphinogold(I) complexes [19,35].

Apoptosis is strongly induced by the most promising iodidogold(I) complex **6b**. This is in line with previous reports about the induction of apoptosis by auranofin (**1a**) and complex **1b** in tumor cells [12,29]. The downregulation of Mcl-1 was described before in association with apoptosis induction in FLO-1 and SK-GT-4 cells [37]. In contrast to the described G2/M arrest of HepG2 hepatoma cells caused by **1b**, the new complexes **6b**, **6d**, and **6i** exhibited no cell cycle arrest in EAC cells. Instead, high sub-G1 levels generated by these complexes indicate a strong preference to induce cell death. The observed suppression of cyclin D1 and *c*-Myc by the complexes **6b**, **6d**, and **6i** might prohibit the ability of treated cells to enter the proper cell cycle, including mitosis, and directly paves the way to the induction of cell death instead. Both factors are relevant for EAC progression in FLO-1 cells [38,39]. In addition, the suppression of *c*-Myc and/or cyclin D1 is important for the treatment of other cancer diseases, which broadens the therapeutic scope of the newly discovered gold complexes [40,41].

The suppression of the formation of colonies and spheroids by EAC cells by complexes **6b**, **6d**, and **6i** is another positive attribute. The downregulation of the stem cell marker CD44 is a hint at an efficient targeting of esophageal cancer stem-like cells (CSCs) and the inhibition of mesenchymal features of EAC cells associated with metastasis formation [42]. EMT reversal in FLO-1 and SK-GT-4 EAC cells can also suppress paracrine effects and the production of exosomes [43].

Since platinum complexes are crucial components of currently applied clinical therapies of EAC, future studies with gold(I)–NHC complexes will probably shed more light on common and distinctive modes of action of platinum and gold complexes. The binding to cysteine and selenocysteine proteins (e.g., thioredoxin reductase) may play a role in the mode of action of the new iodidogold(I) complexes. The relevance of selenium was also highlighted by the suppression of selenium-binding protein 1 as a sign of EAC formation [44]. The combination of gold complexes with HDAC inhibitors might be promising when considering the known effects of HDAC inhibition on thioredoxin and thioredoxin-interacting protein in EAC [45,46]. In addition, aurora kinase inhibitors revealed promising anticancer effects in combination with docetaxel or cisplatin in p53-mutant FLO-1 cells. Thus, they might also be suitable combination partners for the treatment of EAC together with active iodidogold(I)–NHC complexes [47,48]. *c*-Myc inhibitors might also be suitable combination partners [40]. Moreover, our groups have recently identified highly active *c*-Myb inhibitors [49,50,51]. This transcription factor was found to be upregulated in EAC and crucial for the immune escape of EAC cells via the miR-145-5p/SPOP/PD-L1 axis [52,53,54]. Hence, a combination of **6b** with a potent *c*-Myb inhibitor seems worth being investigated in EAC cells.

## 4. Materials and Methods

### 4.1. General Procedures

Column chromatography: silica gel 60 (230–400 mesh, Merck, Darmstadt, Germany). Melting points (uncorrected), Electrothermal 9100 (Thermo Fisher Scientific, Geel, Belgium); IR spectra, Perkin-Elmer Spectrum One FT-IR spectrophotometer with ATR sampling unit (Perkin-Elmer, Rodgau, Germany); NMR spectra, Bruker Avance 300/500 spectrometer (Bruker, Billerica, MA, USA); chemical shifts (δ) are given in parts per million (ppm) downfield from tetramethylsilane as internal standard; mass spectra, Thermo Finnigan MAT 8500 (EI, Thermo Finnigan, San Jose, CA, USA).

### 4.2. Materials

Starting compounds and reagents were obtained from abcr (Karlsruhe, Germany), Sigma-Aldrich (Darmstadt, Germany) and TCI (Zwijndrecht, Belgium). Compound **1b** was prepared according to a literature procedure, and analytical data of the newly prepared compound were in line with published data [24]. The known intermediates **3k–m** and **4k–m**, **5k**, and **5o** were also prepared following procedures from the literature [25,26,27].

### 4.3. Synthesis

#### 4.3.1. Synthesis of Imidazoles 3—Typical Procedure

Benzaldehyde derivatives (1.00 equiv.) were dissolved in EtOH (15.0 mL/mmol). Then, 2 M EtNH2/THF (5.00 equiv.) and AcOH (10.0 equiv.) were added, and the reaction mixture was stirred under reflux for 2 h. After cooling to room temperature, the anisyl-TosMIC reagent **2** (1.50 equiv.) was dissolved in DME (5.00 mL/mmol) and added to the reaction mixture together with K2CO3 (4.00 equiv.). The reaction mixture was then stirred again under reflux for 5 h. The solvent was evaporated, and the residue was taken up in ethyl acetate and water. The organic phase was washed with brine, dried over MgSO4, and filtered, and the filtrate was removed in vacuum. The residue was purified by column chromatography (silica gel 60). The products were obtained as yellow to off-white solids or oils with yields of 55–100%.

**3a**: yield: 100% (quant.); *R*_f_ = 0.22 (ethyl acetate); ^1^H NMR (500 MHz, CDCl_3_) δ 7.62 (s, 1H, *H*^ar^), 7.43 (t, *J* = 1.9 Hz, 1H, *H*^ar^), 7.36 (dt, *J* = 8.9 Hz, 2.6 Hz, 2H, *H*^ar^), 7.23 (d, *J* = 1.9 Hz, 2H, *H*^ar^), 6.80 (dt, *J* = 8.9 Hz, 2.6 Hz, 2H, *H*^ar^), 3.84 (q, *J* = 7.3 Hz, 2H, NC*H*_2_), 3.78 (s, 3H, OC*H*_3_), 1.30 (t, *J* = 7.3 Hz, 3H, CH_2_C*H*_3_); ^13^C NMR (126 MHz, CDCl_3_) δ 162.84 (d, *J_C-F_* = 248.8 Hz, C^ar^), 158.57 (N*C*N), 139.25 (C^ar^), 136.55 (C^ar^), 135.59 (C^ar^), 134.20 (C^ar^), 132.70 (d, *J_C-F_* = 8.6 Hz, C^ar^), 129.12 (C^ar^), 128.81 (C^ar^), 127.99 (C^ar^), 126.62 (C^ar^), 124.39 (C^ar^), 116.23 (d, *J_C-F_* = 21.7 Hz, C^ar^), 113.81 (C=C), 55.22 (O*C*H_3_), 40.31 (N*C*H_2_), 16.42 (CH_2_*C*H_3_).

**3b**: yield: 100% (quant.); *R*_f_ = 0.21 (ethyl acetate); ^1^H NMR (500 MHz, CDCl_3_) δ 7.61 (s, 1H, *H*^ar^), 7.43 (dq, *J* = 8.8, 2.3 Hz, 2H, *H*^ar^), 7.38–7.34 (m, 2H, *H*^ar^), 7.28 (d, *J* = 2.0 Hz, 1H, *H*^ar^), 6.78–6.75 (m, 2H, *H*^ar^), 3.82 (q, *J* = 7.3 Hz, 2H, NC*H*_2_), 3.76 (s, 3H, OC*H*_3_), 1.28–1.25 (m, 3H CH_2_C*H*_3_); ^13^C NMR (126 MHz, CDCl_3_) δ 158.31 (N*C*N), 138.56 (C^ar^), 136.18 (C^ar^), 134.64 (C^ar^), 132.15 (C^ar^), 132.11 (C^ar^), 129.58 (C^ar^), 129.41 (C^ar^), 127.87 (C^ar^), 127.60 (C^ar^), 127.16 (C^ar^), 125.97 (C^ar^), 113.52 (C=C) 55.19 (O*C*H_3_), 40.14 (N*C*H_2_), 16.46 (CH_2_*C*H_3_).

**3c**: yield: 79%; *R*_f_ = 0.20 (ethyl acetate); ^1^H NMR (500 MHz, CDCl_3_) δ 7.60–7.58 (m, 2H, *H*^ar^), 7.38–7.34 (m, 2H, *H*^ar^), 7.23–7.19 (m, 2H, *H*^ar^), 6.78–6.76 (m, 2H, *H*^ar^), 3.85–3.81 (m, 2H, NC*H*_2_), 3.77 (s, 3H, OC*H*_3_), 1.29–1.25 (m, 3H CH_2_C*H*_3_); ^13^C NMR (126 MHz, CDCl_3_) δ 158.33 (N*C*N), 136.22 (C^ar^), 132.42 (C^ar^), 132.36 (C^ar^), 132.12 (C^ar^), 127.88 (C^ar^), 127.59 (C^ar^), 122.86 (C^ar^), 114.50, 113.67 (C=C), 55.19 (O*C*H_3_), 40.15 (N*C*H_2_), 16.51 (CH_2_*C*H_3_), 16.47 (CH_2_*C*H_3_).

**3d**: yield: 79%; *R*_f_ = 0.18 (ethyl acetate); ^1^H NMR (500 MHz, CDCl_3_) δ 7.79–7.75 (m, 2H, *H*^ar^), 7.38–7.34 (m, 2H, *H*^ar^), 7.09–7.04 (m, 2H, *H*^ar^), 6.79–6.75 (m, 2H, *H*^ar^), 3.82 (q, *J* = 7.3 Hz, 2H, NC*H*_2_), 3.76 (s, 3H, OC*H*_3_), 1.25 (t, *J* = 7.3 Hz, 4H CH_2_C*H*_3_); ^13^C NMR (126 MHz, CDCl_3_) δ 158.33 (N*C*N), 138.29 (C^ar^), 136.25 (C^ar^), 132.56 (C^ar^), 130.63 (C^ar^), 127.91 (C^ar^), 127.13 (C^ar^), 126.08 (C^ar^), 113.68 (C=C), 55.20 (O*C*H_3_), 40.15 (N*C*H_2_), 16.48 (CH_2_*C*H_3_).

**3e**: yield: 64%; *R*_f_ = 0.24 (ethyl acetate); ^1^H NMR (500 MHz, CDCl_3_) δ 7.61 (s, 1H, *H*^ar^), 7.38–7.34 (m, 2H, *H*^ar^), 6.87 (dq, *J* = 6.0, 1.6 Hz, 2H, *H*^ar^), 6.80–6.77 (m, 2H, *H*^ar^), 3.86 (q, *J* = 7.3 Hz, 2H, NC*H*_2_), 3.78 (s, 3H, OC*H*_3_), 1.29 (t, *J* = 7.3 Hz, 3H CH_2_C*H*_3_); ^13^C NMR (126 MHz, CDCl_3_) δ 164.23 (dd, *J_C-F_* = 250,2, 13.2 Hz), 162.24 (d, *J_C-F_* = 13.2 Hz), 158.54 (N*C*N), 139.13 (C^ar^), 136.54 (C^ar^), 134.30 (t, *J_C-F_* = 10.2 Hz, C^ar^), 128.04 (C^ar^), 126.71 (C^ar^), 113.84–113.66 (m, C^ar^), 104.25 (t, *J_C-F_* = 25.2 Hz), 55.21 (O*C*H_3_), 40.29 (N*C*H_2_), 16.42 (CH_2_*C*H_3_).

**3f**: yield: 55%; *R*_f_ = 0.29 (ethyl acetate); ^1^H NMR (500 MHz, CDCl_3_) δ 7.62 (s, 1H, *H*^ar^), 7.43 (t, *J* = 1.9 Hz, 1H, *H*^ar^), 7.38–7.33 (m, 2H, *H*^ar^), 7.23 (d, *J* = 1.9 Hz, 2H, *H*^ar^), 6.83–6.77 (m, 2H, *H*^ar^), 3.84 (q, *J* = 7.3 Hz, 2H, NC*H*_2_), 3.78 (s, 3H, OC*H*_3_), 1.30 (t, *J* = 7.3 Hz, 3H CH_2_C*H*_3_); ^13^C NMR (126 MHz, CDCl_3_) δ 158.57 (N*C*N), 139.25 (C^ar^), 136.55 (C^ar^), 135.59 (C^ar^), 134.20 (C^ar^), 129.12 (C^ar^), 128.81 (C^ar^), 127.99 (C^ar^), 126.62 (C^ar^), 124.39 (C^ar^), 113.81 (C=C), 55.22 (O*C*H_3_), 40.31 (N*C*H_2_), 16.42 (CH_2_*C*H_3_).

**3g**: yield: 64%; *R*_f_ = 0.25 (ethyl acetate); ^1^H NMR (500 MHz, CDCl_3_) δ 7.74 (t, *J* = 1.8 Hz, 1H, *H*^ar^), 7.61 (s, 1H, *H*^ar^), 7.43 (d, *J* = 1.8 Hz, 2H, *H*^ar^), 7.39–7.34 (m, 2H, *H*^ar^), 6.84–6.77 (m, 2H, *H*^ar^), 3.84 (q, *J* = 7.3 Hz, 2H, NC*H*_2_), 3.78 (s, 3H, OC*H*_3_), 1.30 (t, *J* = 7.3 Hz, 3H CH_2_C*H*_3_); ^13^C NMR (126 MHz, CDCl_3_) δ 158.57 (N*C*N), 139.28 (C^ar^), 136.54 (C^ar^), 134.77 (C^ar^), 134.23 (C^ar^), 132.38 (C^ar^), 127.97 (C^ar^), 126.61 (C^ar^), 124.17 (C^ar^), 123.44 (C^ar^), 113.82 (C=C), 55.23 (O*C*H_3_), 40.31 (N*C*H_2_), 16.42 (CH_2_*C*H_3_).

**3h**: yield: 76%; *R*_f_ = 0.27 (ethyl acetate); *υ*_max_(ATR)/cm^−1^ 2935, 2836, 1612, 1582, 1559, 1518, 1500, 1462, 1414, 1338, 1317, 1294, 1242, 1167, 1137, 1105, 1024, 953, 865, 835, 815, 798, 765, 743, 663; ^1^H NMR (300 MHz, CDCl_3_) δ 7.53 (s, 1H, *H*^ar^), 7.39 (d, *J* = 8.9 Hz, 2H, *H*^ar^), 6.9–6.8 (m, 2H, *H*^ar^), 6.77 (s, 1H, *H*^ar^), 6.70 (d, *J* = 8.9 Hz, 2H, *H*^ar^), 3.89 (s, 3H, OC*H*_3_), 3.8–3.7 (m, 5H, NC*H*_2_, OC*H*_3_), 3.69 (s, 3H, OC*H*_3_), 1.22 (t, *J* = 7.3 Hz, 3H, CH_2_C*H*_3_); ^13^C NMR (75.5 MHz, CDCl_3_) δ 157.9 (C^ar^), 149.1 (C^ar^), 137.6 (C^ar^), 135.4 (C^ar^), 131.9 (C^ar^), 128.4 (C^ar^), 127.4 (C^ar^), 127.0 (C^ar^), 123.2 (C^ar^), 114.3 (C^ar^), 113.6 (C^ar^), 113.4 (C^ar^), 111.4 (C^ar^), 55.8 (O*C*H_3_), 55.7 (O*C*H_3_), 55.0 (O*C*H_3_), 39.8 (N*C*H_2_), 16.3 (CH_2_*C*H_3_); *m/z* (%) 338 (100) [M^+^], 323 (27), 308 (23).

**3i**: yield: 70%; *R*_f_ = 0.28 (ethyl acetate); *υ*_max_(ATR)/cm^−1^ 2976, 2938, 2838, 1615, 1580, 1520, 1499, 1462, 1442, 1422, 1339, 1299, 1267, 1245, 1213, 1174, 1132, 1105, 1024, 953, 880, 835, 818, 799, 761, 744, 706, 662; ^1^H NMR (300 MHz, CDCl_3_) δ 7.54 (s, 1H, *H*^ar^), 7.35 (d, *J* = 9.0 Hz, 2H, *H*^ar^), 7.1–7.0 (m, 3H, *H*^ar^), 6.72 (d, *J* = 9.0 Hz, 2H, *H*^ar^), 3.89 (s, 3H, OC*H*_3_), 3.76 (q, *J* = 7.3 Hz, 2H, NC*H*_2_), 3.71 (s, 3H, OC*H*_3_), 1.22 (t, *J* = 7.3 Hz, 3H, CH_2_C*H*_3_); ^13^C NMR (75.5 MHz, CDCl_3_) δ 158.1 (C^ar^), 153.8–150.5 (m, C^ar^), 147.8 (C^ar^), 138.2 (C^ar^), 135.7 (C^ar^), 132.0 (C^ar^), 127.9–127.3 (m, C^ar^), 127.0 (C^ar^), 125.7 (C^ar^), 123.4 (C^ar^), 118.4–118.2 (m, C^ar^), 114.4 (C^ar^), 113.6–113.4 (m, C^ar^), 56.0 (O*C*H_3_), 55.0 (O*C*H_3_), 39.9 (N*C*H_2_), 16.3 (CH_2_*C*H_3_); *m/z* (%) 326 (100) [M^+^], 311 (43), 119 (17).

**3j**: yield: 50%; *R*_f_ = 0.31 (ethyl acetate); *υ*_max_(ATR)/cm^−1^ 3117, 3066, 3019, 2981, 2942, 2902, 2839, 1612, 1577, 1560, 1512, 1485, 1462, 1386, 1372, 1343, 1308, 1285, 1242, 1194, 1173, 1147, 1118, 1104, 1056, 1043, 1025, 1018, 951, 900, 834, 819, 808, 796, 744, 703, 676, 653; ^1^H NMR (300 MHz, CDCl_3_) δ 7.55 (s, 1H, *H*^ar^), 7.51 (s, 1H, *H*^ar^), 7.4–7.3 (m, 2H, *H*^ar^), 7.3–7.2 (m, 1H, *H*^ar^), 7.0–6.9 (m, 1H, *H*^ar^), 6.8–6.7 (m, 2H, *H*^ar^), 3.92 (s, 3H, OC*H*_3_), 3.9–3.7 (m, 5H, NC*H*_2_, OC*H*_3_), 1.3–1.2 (m, 3H, CH_2_C*H*_3_); ^13^C NMR (75.5 MHz, CDCl_3_) δ 159.7 (C^ar^), 158.2 (C^ar^), 156.0 (C^ar^), 138.3 (C^ar^), 135.8 (C^ar^), 135.5 (C^ar^), 135.3 (C^ar^), 132.0 (C^ar^), 131.3 (C^ar^), 127.6 (C^ar^), 127.2 (C^ar^), 125.5 (C^ar^), 124.5 (C^ar^), 114.4 (C^ar^), 113.6 (C^ar^), 113.5 (C^ar^), 112.1 (C^ar^), 112.0 (C^ar^), 56.2 (O*C*H_3_), 55.1 (O*C*H_3_), 40.0 (N*C*H_2_), 16.4 (CH_2_*C*H_3_); *m/z* (%) 388 (100) [M^+^], 386 (100) [M^+^], 375 (25), 373 (27), 308 (92), 293 (34), 119 (42).

**3n**: yield: 64%; *R*_f_ = 0.30 (ethyl acetate); *υ*_max_(ATR)/cm^−1^ 2959, 2930, 2844, 1611, 1595, 1557, 1541, 1513, 1463, 1445, 1425, 1408, 1393, 1355, 1334, 1315, 1295, 1273, 1247, 1236, 1197, 1182, 1155, 1108, 1073, 1024, 1000, 955, 873, 856, 844, 826, 797, 754, 742, 712, 687, 660; ^1^H NMR (300 MHz, CDCl_3_) δ 7.57 (s, 1H, *H*^ar^), 7.40 (d, *J* = 9.0 Hz, 2H, *H*^ar^), 7.34 (s, 1H, *H*^ar^), 6.8–6.7 (m, 3H, *H*^ar^), 3.90 (s, 3H, OC*H*_3_), 3.82 (q, *J* = 7.3 Hz, 2H, NC*H*_2_), 3.76 (s, 3H, OC*H*_3_), 3.73 (s, 3H, OC*H*_3_), 1.29 (t, *J* = 7.3 Hz, 3H, CH_2_C*H*_3_); ^13^C NMR (75.5 MHz, CDCl_3_) δ 158.3 (C^ar^), 152.7 (C^ar^), 149.1 (C^ar^), 138.3 (C^ar^), 135.9 (C^ar^), 132.3 (C^ar^), 132.0 (C^ar^), 128.8 (C^ar^), 127.7 (C^ar^), 127.1 (C^ar^), 125.4 (C^ar^), 115.4 (C^ar^), 114.4 (C^ar^), 113.6 (C^ar^), 92.7 (C^ar^), 60.5 (O*C*H_3_), 56.0 (O*C*H_3_), 55.1 (O*C*H_3_), 40.1 (N*C*H_2_), 16.5 (CH_2_*C*H_3_); *m/z* (%) 464 (1) [M^+^], 330 (2), 239 (12), 210 (12), 135 (83), 57 (98), 43 (100).

#### 4.3.2. Synthesis of Diethylimidazolium Iodides 4—Typical Procedure

Imidazoles **3** (1.00 equiv.) were dissolved in acetonitrile (60.0 mL/mmol) and treated with ethyl iodide (55.0 equiv.). The reaction mixture was stirred at 85 °C for 24–70 h. The reaction was concentrated in vacuum, and the residue was dissolved in a small amount of CH2Cl2 and dropped into Et_2_O, leading to the precipitation of the product. The solvent was decanted, and the residue was dried in vacuum. The products were obtained as brown or off-white solids or oils in yields of 74–91%.

**4a**: yield: 100% (quant.); ^1^H NMR (500 MHz, CDCl_3_) δ 10.28 (s, 1H, NC*H*N), 7.33–7.29 (m, 2H, *H*^ar^), 7.22–7.18 (m, 2H, *H*^ar^), 7.11–7.07 (m, 2H, *H*^ar^), 6.91–6.87 (m, 2H, *H*^ar^), 4.27 (dq, *J* = 7.3, 4.7 Hz, 4H, NC*H*_2_), 3.79 (s, 3H, OC*H*_3_), 1.50 (dt, *J* = 7.4, 1.6 Hz, 6H, CH_2_C*H*_3_); ^13^C NMR (126 MHz, CDCl_3_) δ 163.61 (d, *J_C-F_* = 252.0 Hz, C^ar^), 161.02 (N*C*N), 132.78 (d, *J_C-F_* = 8.6 Hz, C^ar^), 131.98 (C^ar^), 116.62 (d, *J_C-F_* = 21.8 Hz, C^ar^), 114.78 (C=C), 55.41 (O*C*H_3_), 43.48 (vd, *J* = 14.5 Hz, N*C*H_2_), 15.75 (CH_2_*C*H_3_).

**4b**: yield: 85%; ^1^H NMR (500 MHz, CDCl_3_) δ 10.29 (s, 1H, NC*H*N), 7.38–7.35 (m, 2H, *H*^ar^), 7.28–7.25 (m, 2H, *H*^ar^), 7.22–7.19 (m, 2H, *H*^ar^), 6.91–6.87 (m, 2H, *H*^ar^), 4.30–4.22 (m, 4H, NC*H*_2_), 3.79 (s, 3H, OC*H*_3_), 1.50 (dt, *J* = 7.3, 3.9 Hz, 6H, CH_2_C*H*_3_); ^13^C NMR (126 MHz, CDCl_3_) δ 161.08 (N*C*N), 136.71 (C^ar^), 132.00 (d, *J* = 2.3 Hz, C^ar^), 129.64 (C^ar^), 123.60 (C^ar^), 116.35 (C^ar^), 114.83 (C=C), 55.42 (O*C*H_3_), 43.57 (vd, *J* = 22.7 Hz, N*C*H_2_), 15.78 (CH_2_*C*H_3_).

**4c**: yield: 91%; ^1^H NMR (500 MHz, CDCl_3_) δ 10.30 (s, 1H, NC*H*N), 7.54–7.51 (m, 2H, *H*^ar^), 7.22–7.18 (m, 4H, *H*^ar^), 6.91–6.89 (m, 2H, *H*^ar^), 4.29–4.25 (m, 4H, NC*H*_2_), 3.79 (s, 3H, OC*H*_3_), 1.52–1.48 (m, 6H, CH_2_C*H*_3_); ^13^C NMR (126 MHz, CDCl_3_) δ 161.09 (N*C*N), 132.60 (C^ar^), 132.17 (C^ar^), 132.00 (C^ar^), 131.92 (C^ar^), 125.03 (C^ar^), 124.09 (C^ar^), 114.84 (C=C), 114.74 (C=C), 55.42 (O*C*H_3_), 43.66 (N*C*H_2_), 43.47 (N*C*H_2_), 15.78 (CH_2_*C*H_3_).

**4d**: yield: 80%; ^1^H NMR (500 MHz, CDCl_3_) δ 10.27 (s, 1H, NC*H*N), 7.74–7.71 (m, 2H, *H*^ar^), 7.23–7.17 (m, 2H, *H*^ar^), 7.07–7.02 (m, 2H, *H*^ar^), 6.89 (dd, *J* = 8.8, 3.6 Hz, 2H, *H*^ar^), 4.30–4.23 (m, 4H, NC*H*_2_), 3.79 (s, 3H, OC*H*_3_), 1.66–1.30 (m, 6H, CH_2_C*H*_3_); ^13^C NMR (126 MHz, CDCl_3_) δ 161.08 (N*C*N), 138.50 (C^ar^), 132.16 (C^ar^), 132.01 (C^ar^), 131.92 (C^ar^), 124.65 (C^ar^), 114.84 (C=C), 114.74 (C=C), 55.43 (O*C*H_3_), 43.56 (vd, *J* = 23.6 Hz, N*C*H_2_), 15.79 (CH_2_*C*H_3_).

**4e**: yield: 74%; ^1^H NMR (500 MHz, CDCl_3_) δ 10.27 (s, 1H, NC*H*N), 7.23–7.18 (m, 2H, *H*^ar^), 6.90–6.81 (m, 5H, *H*^ar^), 4.24 (dq, *J* = 23.8, 7.3 Hz, 4H, NC*H*_2_), 3.76 (s, 3H, OC*H*_3_), 1.48 (dt, *J* = 18.7, 7.3 Hz, 6H, CH_2_C*H*_3_); ^13^C NMR (126 MHz, CDCl_3_) δ 169.95 (s, C^ar^), 162,9 (dd, *J_C-F_* = 251.1, 13.2 Hz, C^ar^) 160.58 (N*C*N), 131.71 (C^ar^), 130.92 (s, C^ar^), 128.48 (s, C^ar^), 118.73 (s, C^ar^), 114.61 (C=C), 114.26–113.91 (m, C^ar^), 105.81 (t, *J_C-F_* = 25.0 Hz, C^ar^), 55.44 (O*C*H_3_), 43.71 (vd, *J* = 32.2 Hz, N*C*H_2_), 15.72 (CH_2_*C*H_3_).

**4f**: yield: 70%; ^1^H NMR (500 MHz, CDCl_3_) δ 10.38 (s, 1H, NC*H*N), 7.44 (t, *J* = 1.9 Hz, 1H, *H*^ar^), 7.27–7.23 (m, 4H, *H*^ar^), 6.97–6.93 (m, 2H, *H*^ar^), 4.29 (dq, *J* = 9.3, 7.3 Hz, 4H, NC*H*_2_), 3.83 (s, 3H, OC*H*_3_), 1.57 (t, *J* = 7.3 Hz, 3H, CH_2_C*H*_3_), 1.52 (t, *J* = 7.3 Hz, 3H, CH_2_C*H*_3_); ^13^C NMR (126 MHz, CDCl_3_) δ 161.30 (N*C*N), 136.00 (C^ar^), 132.87 (C^ar^), 131.99 (C^ar^), 130.76 (C^ar^), 129.00 (C^ar^), 128.18 (C^ar^), 115.83 (C^ar^), 114.97 (C=C), 55.46 (O*C*H_3_), 43.71 (vd, *J* = 27.7 Hz, N*C*H_2_), 15.77 (CH_2_*C*H_3_), 15.75 (CH_2_*C*H_3_).

**4g**: yield: 89%; ^1^H NMR (500 MHz, CDCl_3_) δ 10.36 (s, 1H, NC*H*N), 7.74 (t, *J* = 1.8 Hz, 1H, *H*^ar^), 7.42 (s, 2H, *H*^ar^), 7.25–7.22 (m, 2H, *H*^ar^), 6.96–6.92 (m, 2H, *H*^ar^), 4.27 (*p*, *J* = 7.2 Hz, 4H, NC*H*_2_), 3.82 (s, 3H, OC*H*_3_), 1.55 (t, *J* = 7.3 Hz, 3H, CH_2_C*H*_3_), 1.50 (t, *J* = 7.3 Hz, 3H, CH_2_C*H*_3_); ^13^C NMR (126 MHz, CDCl_3_) δ 161.30 (N*C*N), 136.21 (C^ar^), 132.88 (C^ar^), 132.21 (C^ar^), 131.98 (C^ar^), 128.68 (C^ar^), 123.71 (C^ar^), 115.82 (C=C), 114.98 (C=C), 55.46 (O*C*H_3_), 43.70 (vd, *J* = 25.9 Hz, N*C*H_2_), 15.77 (CH_2_*C*H_3_), 15.74 (CH_2_*C*H_3_).

**4h**: yield: 100% (quant.); *υ*_max_(ATR)/cm^−1^ 3438, 3128, 2978, 2937, 2837, 1609, 1596, 1559, 1521, 1507, 1462, 1428, 1386, 1350, 1293, 1250, 1230, 1177, 1139, 1111, 1091, 1019, 883, 840, 805, 765, 740; ^1^H NMR (300 MHz, CDCl_3_) δ 10.03 (s, 1H, *H*^ar^), 7.16 (d, *J* = 8.8 Hz, 2H, *H*^ar^), 6.9–6.8 (m, 4H, *H*^ar^), 6.71 (s, 1H, *H*^ar^), 4.3–4.1 (m, 4H, NC*H*_2_), 3.79 (s, 3H, OC*H*_3_), 3.72 (s, 3H, OC*H*_3_), 3.68 (s, 3H, OC*H*_3_), 1.5–1.3 (m, 6H, CH_2_C*H*_3_); ^13^C NMR (75.5 MHz, CDCl_3_) δ 160.6 (C^ar^), 150.2 (C^ar^), 149.0 (C^ar^), 134.8 (C^ar^), 131.8 (C^ar^), 131.5 (C^ar^), 131.4 (C^ar^), 123.3 (C^ar^), 116.9 (C^ar^), 116.7 (C^ar^), 114.5 (C^ar^), 113.2 (C^ar^), 111.2 (C^ar^), 56.0 (O*C*H_3_), 55.7 (O*C*H_3_), 55.2 (O*C*H_3_), 43.3 (N*C*H_2_), 43.1 (N*C*H_2_), 15.6 (CH_2_*C*H_3_); *m*/*z* (%) 352 (13), 338 (100), 323 (32), 308 (16), 156 (16), 142 (42), 127 (23).

**4i**: yield: 100% (quant.); *υ*_max_(ATR)/cm^−1^ 3452, 3128, 2979, 2935, 2839, 1616, 1601, 1559, 1524, 1508, 1439, 1387, 1351, 1294, 1272, 1251, 1216, 1177, 1133, 1111, 1091, 1041, 1019, 896, 840, 806, 761, 742; ^1^H NMR (300 MHz, CDCl_3_) δ 10.04 (s, 1H, *H*^ar^), 7.16 (d, *J* = 8.8 Hz, 2H, *H*^ar^), 7.1–6.9 (m, 3H, *H*^ar^), 6.83 (d, *J* = 8.8 Hz, 2H, *H*^ar^), 4.3–4.1 (m, 4H, NC*H*_2_), 3.81 (s, 3H, OC*H*_3_), 3.72 (s, 3H, OC*H*_3_), 1.5–1.4 (m, 6H, CH_2_C*H*_3_); ^13^C NMR (75.5 MHz, CDCl_3_) δ 160.8 (C^ar^), 153.4–150.1 (m, C^ar^), 149.1 (C^ar^), 135.0 (C^ar^), 131.9–131.7 (m, C^ar^), 130.3 (C^ar^), 127.9–127.3 (m, C^ar^), 127.3 (C^ar^), 118.0–117.8 (m, C^ar^), 116.9 (C^ar^), 116.3 (C^ar^), 114.5 (C^ar^), 113.7 (C^ar^), 56.1 (O*C*H_3_), 55.2 (O*C*H_3_), 43.3 (N*C*H_2_), 43.2 (N*C*H_2_), 15.6 (CH_2_*C*H_3_), 15.5 (CH_2_*C*H_3_); *m*/*z* (%) 355 (3) [M^+^], 340 (13), 326 (100), 311 (53), 142 (24), 127 (17).

**4j**: yield: 100% (quant.); *υ*_max_(ATR)/cm^−1^ 3437, 3128, 2977, 2935, 2838, 1608, 1593, 1559, 1518, 1497, 1456, 1386, 1350, 1290, 1250, 1176, 1111, 1091, 1050, 1040, 1017, 964, 915, 846, 829, 805, 771, 743, 721, 682, 655; ^1^H NMR (300 MHz, CDCl_3_) δ 10.08 (s, 1H, *H*^ar^), 7.40 (s, 1H, *H*^ar^), 7.3–7.1 (m, 3H, *H*^ar^), 6.9–6.8 (m, 3H, *H*^ar^), 4.2–4.1 (m, 4H, NC*H*_2_), 3.82 (s, 3H, OC*H*_3_), 3.73 (s, 3H, *H*^ar^), 1.5–1.4 (m, 6H, CH_2_C*H*_3_); ^13^C NMR (75.5 MHz, CDCl_3_) δ 160.8 (C^ar^), 160.7 (C^ar^), 157.1 (C^ar^), 135.1 (C^ar^), 134.9 (C^ar^), 134.7 (C^ar^), 132.0 (C^ar^), 131.7 (C^ar^), 131.5 (C^ar^), 131.4 (C^ar^), 130.2 (C^ar^), 118.1 (C^ar^), 116.7 (C^ar^), 116.3 (C^ar^), 114.6 (C^ar^), 114.5 (C^ar^), 112.2 (C^ar^), 112.0 (C^ar^), 56.3 (O*C*H_3_), 55.2 (O*C*H_3_), 43.3 (N*C*H_2_), 43.1 (N*C*H_2_), 15.6 (CH_2_*C*H_3_), 15.5 (CH_2_*C*H_3_); *m*/*z* (%) 418 (3) [M^+^], 416 (3) [M^+^], 402 (17), 400 (16), 388 (63), 386 (67), 373 (37), 371 (34), 308 (43), 142 (100), 127 (53).

**4n**: yield: 100% (quant.); *υ*_max_(ATR)/cm^−1^ 2973, 2937, 2837, 1606, 1549, 1515, 1482, 1462, 1413, 1397, 1351, 1293, 1250, 1177, 1149, 1110, 1091, 1022, 998, 892, 840, 799, 775, 724, 687; ^1^H NMR (300 MHz, CDCl_3_) δ 10.05 (s, 1H, *H*^ar^), 7.3–7.2 (m, 3H, *H*^ar^), 6.9–6.8 (m, 3H, *H*^ar^), 4.3–4.1 (m, 4H, NC*H*_2_), 3.77 (s, 3H, OC*H*_3_), 3.75 (s, 3H, OC*H*_3_), 3.69 (s, 3H, OC*H*_3_), 1.50 (t, *J* = 7.3 Hz, 3H, CH_2_C*H*_3_), 1.43 (t, *J* = 7.3 Hz, 3H, CH_2_C*H*_3_); ^13^C NMR (75.5 MHz, CDCl_3_) δ 160.9 (C^ar^), 152.5 (C^ar^), 150.1 (C^ar^), 135.3 (C^ar^), 132.2 (C^ar^), 131.8 (C^ar^), 131.5 (C^ar^), 129.9 (C^ar^), 122.6 (C^ar^), 116.4 (C^ar^), 115.5 (C^ar^), 114.4 (C^ar^), 114.1 (C^ar^), 92.5 (C^ar^), 60.5 (O*C*H_3_), 56.4 (O*C*H_3_), 55.2 (O*C*H_3_), 43.5 (N*C*H_2_), 43.2 (N*C*H_2_), 15.6 (CH_2_*C*H_3_), 15.4 (CH_2_*C*H_3_); *m*/*z* (%) 464 (1) [M^+^], 330 (2), 239 (12), 210 (12), 135 (83), 57 (98), 43 (100).

#### 4.3.3. Synthesis of Chloridogold(I) Complexes 5—Typical Procedure

Imidazolium salts **4** (1.00 equiv.) were dissolved in CH_2_Cl_2_ (30 mL/mmol) and Ag_2_O (0.60 equiv.) was added. The reaction mixture was stirred at room temperature for 6 h at r.t. in the dark. Thereupon, AuCl(SMe2) (1.10 equiv.) was added, and the reaction mixture was stirred at room temperature for 24 h in the dark. The mixture was filtered through Celite^®^, the filtrate was concentrated in vacuum, and the product was recrystallized from CH_2_Cl_2_/*n*-pentane or CH_2_Cl_2_/*n*-hexane. The products were obtained as colorless or off-white solids or gums in yields of 72–96%.

**5a**: yield: 96%; mp 140 °C; *υ*_max_(ATR)/cm^−1^ 2971, 2839, 1635, 1599, 1574, 1519, 1503, 1459, 1414, 1371, 1344, 1315, 1291, 1247, 1221, 1175, 1157, 1109, 1095, 1055, 1026, 1014, 960, 834, 821, 810, 788, 738, 724, 691, 658; ^1^H NMR (500 MHz, CDCl_3_) δ 7.30–7.16 (m, 2H, *H*^ar^), 7.12–7.08 (m, 2H, *H*^ar^), 7.07–7.03 (m, 2H, *H*^ar^), 6.89–6.85 (m, 2H, *H*^ar^), 4.16 (dq, *J* = 7.2 Hz, 3.8 Hz, 4H, NC*H*_2_), 3.80 (s, 3H, OC*H*_3_), 1.29 (t, *J* = 7.2 Hz, 6H, CH_2_C*H*_3_); ^13^C NMR (125 MHz, CDCl_3_) δ 169.1 (N*C*N), 163.2 (d, *J_C-F_* = 245.7 Hz, C^ar^), 160.4 (C^ar^), 132.6 (d, *J_C-F_* = 8.5 Hz, C^ar^), 131.9 (C^ar^), 131.4 (C^ar^), 130.0 (C^ar^), 124.0 (C^ar^), 119.5 (C^ar^), 116.4 (C^ar^), 116.2 (C^ar^), 114.5 (C=C), 55.42 (O*C*H_3_), 44.49 (N*C*H_2_), 44.43 (N*C*H_2_), 17.05 (CH_2_*C*H_3_); *m*/*z* (%) 568 (34) [M^+^], 566 (100) [M^+^], 521 (83) [M^+^–Cl], 519 (48), 492 (17), 323 (52) [M^+^–AuCl].

**5b**: yield: 84%; mp 186 °C; *υ*_max_(ATR)/cm^−1^ 2994, 2971, 2928, 2838, 1635, 1608, 1591, 1574, 1514, 1489, 1459, 1442, 1406, 1365, 1344, 1315, 1290, 1247, 1174, 1108, 1091, 1056, 1028, 1013, 963, 841, 831, 810, 791, 733, 722, 710, 690; ^1^H NMR (500 MHz, CDCl_3_) δ 7.35–7.31 (m, 2H, *H*^ar^), 7.15–7.12 (m, 2H, *H*^ar^), 7.12–7.08 (m, 2H, *H*^ar^), 6.90–6.83 (m, 2H, *H*^ar^), 4.17 (vdq, *J* = 11 Hz, 7.3 Hz, 4H, NC*H*_2_), 3.80 (s, 3H, OC*H*_3_), 1.29 (t, *J* = 7.1 Hz, 6H, CH_2_C*H*_3_); ^13^C NMR (125 MHz, CDCl_3_) δ 169.4 (N*C*N), 160.5 (C^ar^), 135.7 (C^ar^), 131.9 (C^ar^), 131.8 (C^ar^), 131.5 (C^ar^), 129.8 (C^ar^), 129.4 (C^ar^), 126.5 (C^ar^), 119.4 (C^ar^), 114.6 (C=C), 55.43 (O*C*H_3_), 44.55 (N*C*H_2_), 17.06 (CH_2_*C*H_3_); *m*/*z* (%) 574 (71) [M^+^], 572 (100) [M^+^], 537 (72) [M^+^–Cl], 535 (74), 339 (62) [M^+^–AuCl].

**5c**: yield: 87%; mp. 183 °C; *υ*_max_(ATR)/cm^−1^ 2972, 2828, 2837, 1634, 1606, 1587, 1573, 1513, 1487, 1459, 1405, 1390, 1365, 1314, 1289, 1246, 1174, 1108, 1085, 1071, 1055, 1027, 1009, 963, 840, 829, 809, 789, 736, 723, 704, 690; ^1^H NMR (500 MHz, CDCl_3_) δ 7.51–7.47 (m, 2H, *H*^ar^), 7.12–7.04 (m, 4H, *H*^ar^), 6.87 (dd, *J* = 9.3 Hz, 7.5 Hz, 2H, *H*^ar^), 4.17 (dq, *J* = 12.8 Hz, 7.2 Hz, 4H, NC*H*_2_), 3.80 (s, 3H, OC*H*_3_), 1.29 (dt, *J* = 7.2 Hz, 1.7 Hz, 6H, CH_2_C*H*_3_); ^13^C NMR (125 MHz, CDCl_3_) δ 169.5 (N*C*N), 160.5 (C^ar^), 132.3 (C^ar^), 132.1 (C^ar^), 131.9 (C^ar^), 131.4 (C^ar^), 129.8 (C^ar^), 127.0 (C^ar^), 119.3 (C^ar^), 114.6 (C=C), 114.4 (C=C), 55.43 (O*C*H_3_), 44.56 (N*C*H_2_C), 44.44 (N*C*H_2_), 17.07 (CH_2_*C*H_3_); *m*/*z* (%) 618 (100) [M^+^], 616 (72) [M^+^], 583 (64) [M^+^–Cl], 581 (86) [M^+^–Cl], 383 (42) [M^+^–AuCl], 134 (41).

**5d**: yield: 86%; mp 188 °C; *υ*_max_(ATR)/cm^−1^ 2998, 2970, 2931, 2833, 1635, 1608, 1573, 1512, 1482, 1461, 1414, 1387, 1346, 1315, 1290, 1245, 1175, 1109, 1082, 1061, 1028, 1005, 841, 801, 736, 722, 692; ^1^H NMR (500 MHz, CDCl_3_) δ 7.71–7.66 (m, 2H, *H*^ar^), 7.11–7.08 (m, 2H, *H*^ar^), 6.94–6.91 (m, 2H, *H*^ar^), 6.90–6.86 (m, 2H, *H*^ar^), 4.17 (dq, *J* = 14 Hz, 7.2 Hz, 4H, NC*H*_2_), 3.80 (s, 3H, OC*H*_3_), 1.29 (dt, *J* = 7.2 Hz, 3.2 Hz, 6H, CH_2_C*H*_3_); ^13^C NMR (125 MHz, CDCl_3_) δ 169.5 (N*C*N), 160.5 (C^ar^), 138.3 (C^ar^), 132.1 (C^ar^), 131.9 (C^ar^), 131.4 (C^ar^), 130.0, 127.5 (C^ar^), 119.3 (C^ar^), 114.5 (C=C), 114.4 (C=C), 55.43 (O*C*H_3_), 44.56 (N*C*H_2_), 44.44 (N*C*H_2_), 17.04 (CH_2_*C*H_3_); *m*/*z* (%) 666 (37) [M^+^], 664 (100) [M^+^], 629 (62) [M^+^–Cl], 431 (27) [M^+^–AuCl]

**5e**: yield: 72%; mp 205 °C; *υ*_max_(ATR)/cm^−1^ 3974, 2928, 2840, 1618, 1590, 1516, 1463, 1433, 1416, 1366, 1345, 1326, 1292, 1274, 1252, 1176, 1117, 1092, 1053, 1029, 986, 868, 836, 812, 787, 766, 687; ^1^H NMR (500 MHz, CDCl_3_) δ 7.13–7.08 (m, 2H, *H*^ar^), 6.93–6.88 (m, 2H, *H*^ar^), 6.83 (tt, *J* = 8.8 Hz, 2.3 Hz, 1H, *H*^ar^), 6.76–6.72 (m, 2H, *H*^ar^), 4.22 (q, *J* = 7.2 Hz, 2H, NC*H*_2_), 4.15 (q, ^3^*J* = 7.2 Hz, 2H, NC*H*_2_), 3.82 (s, 3H, OC*H*_3_), 1.31 (dt, *J* = 14 Hz, 7.2 Hz, 6H, CH_2_C*H*_3_); ^13^C NMR (125 MHz, CDCl_3_) δ 170.1 (N*C*N), 162.9 (dd, *J_C-F_* = 245.7 Hz, 12.6 Hz, C^ar^), 160.7 (C^ar^), 131.9 (C^ar^), 131.8 (C^ar^), 131.0 (C^ar^), 128.6 (C^ar^), 118.9 (C^ar^), 114.7 (C=C), 113.9 (C=C), 55.47 (O*C*H_3_), 44.73 (N*C*H_2_), 44.52 (N*C*H_2_), 17.07 (CH_2_*C*H_3_); *m*/*z* (%) 576 (33) [M^+^], 574 (100) [M^+^], 539 (67) [M^+^–Cl], 341 (42) [M^+^–AuCl].

**5f**: yield: 87%; mp 260 °C; *υ*_max_(ATR)/cm^−1^ 3064, 2974, 2928, 2837, 1640, 1609, 1583, 1558, 1515, 1461, 1440, 1416, 1378, 1345, 1309, 1293, 1254, 1176, 1132, 1119, 1103, 1032, 974, 867, 851, 831, 803, 746, 685; ^1^H NMR (500 MHz, CDCl_3_) δ 7.37 (t, *J* = 1.9 Hz, 1H, *H*^ar^), 7.13–7.09 (m, 4H, *H*^ar^), 6.94–6.88 (m, 2H, *H*^ar^), 4.17 (vdq, *J* = 20 Hz, 7.2 Hz, 4H, NC*H*_2_), 3.82 (s, 3H, OC*H*_3_), 1.31 (vdt, *J* = 17 Hz, 7.1 Hz, 6H, CH_2_C*H*_3_); ^13^C NMR (125 MHz, CDCl_3_) δ 170.1 (N*C*N), 160.7 (C^ar^), 135.7 (C^ar^), 132.1 (C^ar^), 131.8 (C^ar^), 131.0 (C^ar^), 129.8 (C^ar^), 128.9 (C^ar^), 128.2 (C^ar^), 118.7 (C^ar^), 114.7 (C=C), 55.48 (O*C*H_3_), 44.73 (N*C*H_2_), 44.53 (N*C*H_2_), 17.14 (CH_2_*C*H_3_), 17.03 (CH_2_*C*H_3_); *m*/*z* (%) 610 (32) [M^+^], 608 (100) [M^+^], 606 (97) [M^+^], 573 (33) [M^+^–Cl], 571 (74) [M^+^–Cl], 569 (46), 375 (26) [M^+^–AuCl], 373 (41) [M^+^–AuCl].

**5g**: yield: 92%; mp 266 °C; *υ*_max_(ATR)/cm^−1^ 3068, 2972, 2928, 2840, 1604, 1578, 1542, 1514, 1461, 1412, 1376, 1345, 1293, 1254, 1176, 1032, 972, 865, 846, 808, 751, 736, 684; ^1^H NMR (500 MHz, CDCl_3_) δ 7.67 (t, *J* = 1.7 Hz, 1H, *H*^ar^), 7.30 (d, *J* = 1.7 Hz, 2H, *H*^ar^), 7.14–7.07 (m, 2H, *H*^ar^), 6.95–6.89 (m, 2H, *H*^ar^), 4.16 (vdq, *J* = 18.6 Hz, 7.0 Hz, 4H, NC*H*_2_), 3.82 (s, 3H, OC*H*_3_), 1.31 (vdt, *J* = 20 Hz, 7.2 Hz, 6H, CH_2_C*H*_3_); ^13^C NMR (125 MHz, CDCl_3_) δ 170.0 (N*C*N), 160.7 (C^ar^), 135.2 (C^ar^), 132.2 (C^ar^), 131.9 (C^ar^), 131.4 (C^ar^), 128.1 (C^ar^), 123.5 (C^ar^), 119.7 (C^ar^), 115.7 (C=C), 55.49 (O*C*H_3_), 44.72 (N*C*H_2_), 44.53 (N*C*H_2_), 17.15 (CH_2_*C*H_3_), 17.03 (CH_2_*C*H_3_); *m*/*z* (%) 698 (75) [M^+^], 696 (100) [M^+^], 694 (44) [M^+^], 663 (35) [M^+^–Cl], 661 (87) [M^+^–Cl], 650 (68), 463 (30) [M^+^–AuCl], 134 (24).

**5h**: yield: 94%; mp > 100 °C (dec.); *υ*_max_(ATR)/cm^−1^ 2963, 2934, 2836, 1610, 1597, 1520, 1506, 1461, 1417, 1377, 1346, 1324, 1291, 1251, 1171, 1139, 1110, 1089, 1022, 974, 886, 839, 809, 764, 737, 694; ^1^H NMR (300 MHz, CDCl_3_) δ 7.2–7.1 (m, 2H, *H*^ar^), 6.9–6.7 (m, 4H, *H*^ar^), 6.61 (s, 1H, *H*^ar^), 4.2–4.1 (m, 4H, NC*H*_2_), 3.85 (3 H, s, OC*H*_3_), 3.78 (3 H, s, OC*H*_3_), 3.73 (3 H, s, OC*H*_3_), 1.4–1.2 (m, 6H, CH_2_C*H*_3_); ^13^C NMR (75.5 MHz, CDCl_3_) δ 168.7 (N*C*N), 160.2 (C^ar^), 149.7 (C^ar^), 148.9 (C^ar^), 131.9 (C^ar^), 131.7 (C^ar^), 130.9 (C^ar^), 123.3 (C^ar^), 120.1(C^ar^), 119.9 (C^ar^), 114.4 (C^ar^), 114.3 (C^ar^), 113.3 (C^ar^), 111.2 (C^ar^), 55.9 (O*C*H_3_), 55.8 (O*C*H_3_), 55.3 (O*C*H_3_), 44.3 (N*C*H_2_), 44.2 (N*C*H_2_), 17.0 (CH_2_*C*H_3_), 16.9 (CH_2_*C*H_3_); *m*/*z* (%) 600 (35) [M^+^], 598 (100) [M^+^], 563 (23) [M^+^–Cl], 561 (17) [M^+^–Cl], 365 (26), 50 (24).

**5i**: yield: 94%; mp >120 °C (dec.); *υ*_max_(ATR)/cm^−1^ 2977, 2935, 2840, 1617, 1599, 1575, 1522, 1506, 1459, 1434, 1405, 1380, 1342, 1291, 1272, 1249, 1180, 1135, 1108, 1090, 1048, 1022, 976, 896, 872, 840, 827, 808, 781, 761, 736, 696; ^1^H NMR (300 MHz, CDCl_3_) δ 7.08 (d, *J* = 8.8 Hz, 2H, *H*^ar^), 6.9–6.8 (m, 5H, *H*^ar^), 4.2–4.1 (m, 4H, NC*H*_2_), 3.86 (s, 3H, OC*H*_3_), 3.79 (s, 3H, OC*H*_3_), 1.4–1.3 (m, 6H, CH_2_C*H*_3_); ^13^C NMR (75.5 MHz, CDCl_3_) δ 169.0 (N*C*N), 160.3 (C^ar^), 150.6–153.3 (m, C^ar^), 148.3 (C^ar^), 131.8–131.7 (m, C^ar^), 131.2 (C^ar^), 126.9–126.8 (m, C^ar^), 120.3 (C^ar^), 119.4 (C^ar^), 118.3–118.0 (m, C^ar^), 114.5–114.3 (m, C^ar^), 113.4 (C^ar^), 56.1 (O*C*H_3_), 55.3 (O*C*H_3_), 44.3 (N*C*H_2_), 16.9 (CH_2_*C*H_3_); *m*/*z* (%) 588 (34) [M^+^], 586 (100) [M^+^], 551 (34) [M^+^–Cl], 549 (31) [M^+^–Cl], 353 (44) [M^+^–AuCl], 50 (26).

**5j**: yield: 73%; *υ*_max_(ATR)/cm^−1^ 2969, 2837, 1608, 1593, 1516, 1495, 1460, 1411, 1378, 1345, 1288, 1249, 1176, 1109, 1089, 1050, 1021, 888, 848, 832, 809, 721, 682; ^1^H NMR (300 MHz, CDCl_3_) δ 7.40 (1 H, s, *H*^ar^), 7.1–7.0 (m, 3H, *H*^ar^), 6.9–6.8 (m, 3H, *H*^ar^), 4.2–4.1 (m, 4H, NC*H2*), 3.87 (s, 3H, OC*H*_3_), 3.79 (s, 3H, OC*H*_3_), 1.3–1.2 (m, 6H, CH_2_C*H*_3_); ^13^C NMR (75.5 MHz, CDCl_3_) δ 169.0 (N*C*N), 160.3 (C^ar^), 156.7 (C^ar^), 135.1 (C^ar^), 131.9 (C^ar^), 131.8 (C^ar^), 131.3 (C^ar^), 131.0 (C^ar^), 129.3 (C^ar^), 121.2 (C^ar^), 119.4 (C^ar^), 114.4 (C^ar^), 114.3 (C^ar^), 112.0 (C^ar^), 111.8 (C^ar^), 56.3 (O*C*H_3_), 55.3 (O*C*H_3_), 44.3 (N*C*H_2_), 17.0 (CH_2_*C*H_3_), 16.9 (CH_2_*C*H_3_); *m*/*z* (%) 648 (66) [M^+^], 646 (48) [M^+^], 611 (20) [M^+^–Cl], 570 (36), 568 (100), 533 (28), 531 (26), 335 (41), 135 (24), 50 (36).

**5l**: yield: 75%; mp >110 °C (dec.); *υ*_max_(ATR)/cm^−1^ 2973, 2938, 2833, 1608, 1563, 1515, 1492, 1461, 1411, 1319, 1293, 1252, 1175, 1109, 1091, 1043, 1003, 901, 835, 812, 755, 709; ^1^H NMR (300 MHz, CDCl_3_) δ 7.11 (d, *J* = 8.8 Hz, 2H, *H*^ar^), 6.88 (d, *J* = 8.8 Hz, 2H, *H*^ar^), 6.85 (s, 1H, *H*^ar^), 6.54 (s, 1H, *H*^ar^), 4.2–4.1 (m, 4H, NC*H*_2_), 3.85 (s, 3H, OC*H*_3_), 3.80 (s, 3H, OC*H*_3_), 3.70 (s, 3H, OC*H*_3_), 1.34 (t, *J* = 7.2 Hz, 3H, CH_2_C*H*_3_), 1.28 (t, *J* = 7.2 Hz, 3H, CH_2_C*H*_3_); ^13^C NMR (75.5 MHz, CDCl_3_) δ 169.3 (N*C*N), 160.4 (C^ar^), 153.8 (C^ar^), 146.2 (C^ar^), 131.8 (C^ar^), 131.2 (C^ar^), 129.6 (C^ar^), 128.7 (C^ar^), 123.9 (C^ar^), 119.3 (C^ar^), 114.5 (C^ar^), 114.3 (C^ar^), 113.2 (C^ar^), 60.8 (OCH_3_), 56.2 (O*C*H_3_), 55.3 (O*C*H_3_), 44.5 (N*C*H_2_), 44.3 (N*C*H_2_), 17.1 (CH_2_*C*H_3_), 16.9 (CH_2_*C*H_3_); *m*/*z* (%) 634 (36) [M^+^], 632 (59) [M^+^], 586 (100), 567 (33), 549 (34), 353 (52), 134 (33), 50 (100).

**5m**: yield: 84%; mp >100 °C (dec.); *υ*_max_(ATR)/cm^−1^ 2970, 2934, 2833, 1607, 1589, 1555, 1514, 1488, 1461, 1410, 1346, 1319, 1292, 1250, 1176, 1156, 1110, 1090, 1027, 998, 896, 840, 810, 754, 699, 662; ^1^H NMR (300 MHz, CDCl_3_) δ 7.11 (d, *J* = 8.8 Hz, 2H, *H*^ar^), 7.02 (s, 1H, *H*^ar^), 6.89 (d, *J* = 8.8 Hz, 2H, *H*^ar^), 6.57 (s, 1H, *H*^ar^), 4.2–4.1 (m, 4H, NC*H*_2_), 3.84 (s, 3H, OC*H*_3_), 3.80 (s, 3H, OC*H*_3_), 3.69 (s, 3H, OC*H*_3_), 1.4–1.2 (m, 6H, CH_2_C*H*_3_); ^13^C NMR (75.5 MHz, CDCl_3_) δ 169.3 (N*C*N), 160.4 (C^ar^), 153.6 (C^ar^), 147.2 (C^ar^), 131.7 (C^ar^), 131.3 (C^ar^), 129.4 (C^ar^), 126.6 (C^ar^), 124.6 (C^ar^), 119.3 (C^ar^), 118.0 (C^ar^), 114.5 (C^ar^), 113.9 (C^ar^), 60.7 (O*C*H_3_), 56.1 (O*C*H_3_), 55.3 (O*C*H_3_), 44.5 (N*C*H_2_), 44.3 (N*C*H_2_), 17.1 (CH_2_*C*H_3_), 16.9 (CH_2_*C*H_3_); *m*/*z* (%) 678 (100) [M^+^], 676 (73) [M^+^], 643 (67) [M^+^–Cl], 641 (76) [M^+^–Cl], 445 (14) [M^+^–AuCl], 443 (14) [M^+^–AuCl], 50 (50).

**5n**: yield: 50%; mp >100 °C (dec.); *υ*_max_(ATR)/cm^−1^ 2970, 2933, 2835, 1606, 1585, 1513, 1482, 1460, 1408, 1345, 1317, 1291, 1249, 1176, 1153, 1109, 1089, 1025, 997, 894, 839, 800, 752, 692, 659; ^1^H NMR (300 MHz, CDCl_3_) δ 7.23 (s, 1H, *H*^ar^), 7.11 (d, *J* = 8.8 Hz, 2H, *H*^ar^), 6.88 (d, *J* = 8.8 Hz, 2H, *H*^ar^), 6.59 (s, 1H, *H*^ar^), 4.2–4.1 (m, 4H, NC*H*_2_), 3.82 (s, 3H, OC*H*_3_), 3.79 (s, 3H, OC*H*_3_), 3.67 (s, 3H, OC*H*_3_), 1.4–1.2 (m, 6H, CH_2_C*H*_3_); ^13^C NMR (75.5 MHz, CDCl_3_) δ 169.2 (N*C*N), 160.4 (C^ar^), 152.4 (C^ar^), 149.7 (C^ar^), 132.2 (C^ar^), 131.9 (C^ar^), 131.2 (C^ar^), 129.2 (C^ar^), 125.4 (C^ar^), 119.3 (C^ar^), 115.0 (C^ar^), 114.4 (C^ar^), 114.3 (C^ar^), 92.5 (C^ar^), 60.5 (O*C*H_3_), 56.3 (O*C*H_3_), 55.3 (O*C*H_3_), 44.4 (N*C*H_2_), 44.3 (N*C*H_2_), 17.1 (CH_2_*C*H_3_), 16.9 (CH_2_*C*H_3_); *m*/*z* (%) 726 (7) [M^+^], 724 (24) [M^+^], 689 (84) [M^+^–Cl], 660 (36), 563 (42), 533 (83), 142 (34), 50 (100).

#### 4.3.4. Synthesis of Iodidogold(I) Complexes 6—Typical Procedure

Chlorido-Au(I) complexes **5** (1.00 equiv.) were dissolved in acetone (45.0 mL/mmol) and treated with KI (4.00 equiv.). The reaction mixture was stirred at room temperature for 24 h. The solvent was removed in vacuum, and the residue was resuspended in CH_2_Cl_2_. The suspension was filtered through Celite^®^ and a plug of silicate, the filtrate was concentrated in vacuum, and the residue was recrystallized from CH_2_Cl_2_/*n*-pentane or CH_2_Cl_2_/*n*-hexane. The products were obtained as colorless solids in yields of 63–100%.

**6a**: yield: 96%; mp 168 °C; *υ*_max_(ATR)/cm^−1^ 2972, 2931, 2838, 1631, 1599, 1573, 1519, 1503, 1459, 1412, 1378, 1345, 1314, 1291, 1248, 1223, 1175, 1157, 1109, 1093, 1054, 1041, 1026, 958, 836, 821, 811, 739, 723, 688, 658; ^1^H NMR (500 MHz, CDCl_3_) δ 7.22–7.16 (m, 2H, *H*^ar^), 7.12–7.08 (m, 2H, *H*^ar^), 7.08–7.03 (m, 2H, *H*^ar^), 6.89–6.85 (m, 2H, *H*^ar^), 4.16 (vdq, *J* = 7.3 Hz, 3.9 Hz, 4H, NC*H*_2_), 3.80 (s, 3H, OC*H*_3_), 1.31 (vdt, *J* = 7.0 Hz, 1.7 Hz, 6H, CH_2_C*H*_3_); ^13^C NMR (125 MHz, CDCl_3_) δ 179.8 (N*C*N), 163.2 (d, *J_C-F_* = 245.7 Hz, C^ar^), 160.4 (C^ar^), 132.6 (C^ar^), 132.5 (C^ar^), 131.9 (C^ar^), 131.2 (C^ar^), 129.8 (C^ar^), 119.5 (C^ar^), 116.4 (C^ar^), 116.2 (C^ar^), 114.5 (C=C), 55.43 (O*C*H_3_), 44.14 (N*C*H_2_), 17.09 (CH_2_*C*H_3_); *m*/*z* (%) 648 (63) [M^+^], 521 (100) [M^+^–I]; anal. calcd. for C_20_H_21_AuFIN_2_O: C, 37.06; H, 3.27; N, 4.32. Found: C, 37.74; H, 3.31; N, 4.29.

**6b**: yield: 100% (quant.); mp 175 °C; *υ*_max_(ATR)/cm^−1^ 2970, 2828, 2833, 1635, 1607, 1572, 1513, 1489, 1460, 1408, 1377, 1344, 1291, 1248, 1175, 1090, 1025, 1013, 835, 795, 734; ^1^H-NMR (500 MHz, CDCl_3_) δ 7.36–7.32 (m, 2H, *H*^ar^), 7.17–7.12 (m, 2H, *H*^ar^), 7.12–7.08 (m, 2H, *H*^ar^), 6.91–6.85 (m, 2H, *H*^ar^), 4.17 (vdq, *J* = 11 Hz, 7.3 Hz, 4H, NC*H*_2_), 3.80 (s, 3H, OC*H*_3_), 1.31 (t, *J* = 7.4 Hz, 6H, CH_2_C*H*_3_); ^13^C NMR (125 MHz, CDCl_3_) δ 180.1 (N*C*N), 160.5 (C^ar^), 135.7 (C^ar^), 131.9 (C^ar^), 131.8 (C^ar^), 131.3 (C^ar^), 129.7 (C^ar^), 129.4 (C^ar^), 126.5 (C^ar^), 119.4 (C^ar^), 114.6 (C=C), 55.44 (O*C*H_3_), 44.20 (N*C*H_2_), 44.10 (N*C*H_2_), 17.11 (CH_2_*C*H_3_); *m*/*z* (%) 666 (18) [M^+^], 664 (53) [M^+^], 539 (36) [M^+^–I], 537 (100) [M^+^–I]; anal. calcd. for C_20_H_21_AuClIN_2_O: C, 36.14; H, 3.18; N, 4.21. Found: C, 36.81; H, 3.15; N, 4.17.

**6c**: yield: 96%; mp 170 °C; *υ*_max_(ATR)/cm^−1^ 2969, 2930, 2836, 1635, 1607, 1586, 1573, 1513, 1485, 1459, 1440, 1416, 1384, 1369, 1345, 1314, 1291, 1245, 1174, 1109, 1099, 1082, 1069, 1028, 1009, 955, 833, 812, 795, 735, 724, 703, 688, 662; ^1^H NMR (500 MHz, CDCl_3_) δ 7.51–7.47 (m, 2H, *H*^ar^), 7.12–7.06 (m, 4H, *H*^ar^), 6.90–6.84 (m, 2H, *H*^ar^), 4.18 (vdq, *J* = 12.8 Hz, 7.2 Hz, 4H, NC*H*_2_), 3.81 (s, 3H, OC*H*_3_), 1.31 (vdt, *J* = 7.2 Hz, 2.2 Hz, 6H, CH_2_C*H*_3_); ^13^C NMR (125 MHz, CDCl_3_) δ 180.2 (N*C*N), 160.5 (C^ar^), 132.3 (C^ar^), 132.1 (C^ar^), 131.9 (C^ar^), 131.3 (C^ar^), 131.0 (C^ar^), 127.0 (C^ar^), 119.4 (C^ar^), 114.6 (C=C), 114.4 (C=C), 55.44 (O*C*H_3_), 44.21 (N*C*H_2_), 44.10 (N*C*H_2_), 17.11 (CH_2_*C*H_3_); *m*/*z* (%) 710 (53) [M^+^], 708 (54) [M^+^], 660 (23), 583 (92) [M^+^–I], 581 (100) [M^+^–I], 533 (47); anal. calcd. for C_20_H_21_AuBrIN_2_O: C, 33.87; H, 2.98; N, 3.95. Found: C, 34.71; H, 2.94; N, 4.07.

**6d**: yield: 91%; mp 143 °C; *υ*_max_(ATR)/cm^−1^ 2994, 2968, 2928, 2834, 1634, 1607, 1583, 1512, 1482, 1460, 1415, 1385, 1369, 1345, 1314, 1291, 1245, 1174, 1109, 1082, 1061, 1027, 1005, 956, 831, 812, 794, 735, 722, 697; ^1^H NMR (500 MHz, CDCl_3_) δ 7.71–7.68 (m, 2H, *H*^ar^), 7.13–7.07 (m, 2H, *H*^ar^), 6.97–6.91 (m, 2H, *H*^ar^), 6.90–6.83 (m, 2H, *H*^ar^), 4.17 (vdq, *J* = 14.2 Hz, 7.1 Hz, 4H, NC*H*_2_), 3.81 (s, 3H, OC*H*_3_), 1.31 (vdt, *J* = 7.2 Hz, 3.3 Hz, 6H, CH_2_C*H*_3_); ^13^C NMR (125 MHz, CDCl_3_) δ 180.2 (N*C*N), 160.5 (C^ar^), 138.3 (C^ar^), 132.1 (C^ar^), 131.9 (C^ar^), 131.2 (C^ar^), 129.8 (C^ar^), 127.5 (C^ar^), 119.4 (C^ar^), 114.6 (C=C), 114.4 (C=C), 55.44 (O*C*H_3_), 44.21 (N*C*H_2_), 44.09 (N*C*H_2_), 17.13 (CH_2_*C*H_3_), 17.09 (CH_2_*C*H_3_); *m*/*z* (%) 756 (67) [M^+^], 660 (23), 629 (100), 533 (43) [M^+^–I], 502 (20); anal. calcd. for C_20_H_21_AuI_2_N_2_O: C, 31.77; H, 2.80; N, 3.70. Found: C, 32.26; H, 2.83; N, 3.67.

**6e**: yield: 93%; mp 164 °C; *υ*_max_(ATR)/cm^−1^ 2965, 2928, 2833, 1616, 1589, 1513, 1459, 1446, 1432, 115, 1371, 1293, 1273, 1249, 1176, 1122, 1090, 1052, 1030, 1011, 991, 961, 880, 863, 836, 810, 786, 763, 736, 720, 686; ^1^H NMR (500 MHz, CDCl_3_) δ 7.14–7.09 (m, 2H, *H*^ar^), 6.92–6.88 (m, 2H, *H*^ar^), 6.83 (tt, *J* = 8.8 Hz, 2.3 Hz, 1H, *H*^ar^), 6.77–6.71 (m, 2H, *H*^ar^), 4.23 (q, *J* = 7.4 Hz, 2H, NC*H*_2_), 4.16 (q, *J* = 7.3 Hz, 2H, NC*H*_2_), 3.82 (s, 3H, OC*H*_3_), 1.33 (dt, *J* = 14 Hz, 7.2, 6H, CH_2_C*H*_3_); ^13^C NMR (125 MHz, CDCl_3_) δ 181.7 (N*C*N), 162.9 (dd, *J_C-F_* = 252.2 Hz, 13.5 Hz, C^ar^), 160.7 (C^ar^), 131.9 (C^ar^), 131.8 (C^ar^), 131.1 (C^ar^), 128.4 (C^ar^), 119.9 (C^ar^), 114.7 (C=C), 113.9 (C=C), 55.47 (O*C*H_3_), 44.37 (N*C*H_2_), 44.17 (N*C*H_2_), 17.07 (CH_2_*C*H_3_); *m*/*z* (%) 666 (53) [M^+^], 539 (100) [M^+^–I]; anal. calcd. for C_20_H_20_AuF_2_IN_2_O: C, 36.05; H, 3.03; N, 4.20. Found: C, 36.49; H, 3.00; N, 4.26.

**6f**: yield: 93%; mp 163 °C; *υ*_max_(ATR)/cm^−1^ 3078, 2976, 2952, 2931, 2835, 1631, 1605, 1583, 1558, 1512, 1440, 1415, 1379, 1346, 1305, 1292, 1250, 1175, 1126, 1110, 1090, 1051, 1027, 1007, 972, 888, 866, 850, 826, 802, 744, 682; ^1^H NMR (500 MHz, CDCl_3_) δ 7.37 (t, *J* = 1.9 Hz, 1H, 1H, *H*^ar^), 7.15–7.10 (m, 4H, *H*^ar^), 6.91 (d, *J* = 8.4 Hz, 2H, *H*^ar^), 4.17 (vdq, *J* = 20 Hz, 7.4 Hz, 4H, NC*H*_2_), 3.82 (s, 3H, OC*H*_3_), 1.32 (vdt, *J* = 17.8 Hz, 7.1 Hz, 6H, CH_2_C*H*_3_); ^13^C NMR (125 MHz, CDCl_3_) δ 180.5 (N*C*N), 160.7 (C^ar^), 135.6 (C^ar^), 132.0 (C^ar^), 131.9 (C^ar^), 131.0 (C^ar^), 129.7 (C^ar^), 128.9 (C^ar^), 128.1 (C^ar^), 118.8 (C^ar^), 114.7 (C=C), 55.48 (O*C*H_3_), 44.36 (N*C*H_2_), 44.18 (N*C*H_2_), 17.18 (CH_2_*C*H_3_), 17.08 (CH_2_*C*H_3_); *m*/*z* (%) 700 (33) [M^+^], 698 (52) [M^+^], 573 (64) [M^+^–I], 571 (100) [M^+^–I]; anal. calcd. for C_20_H_20_AuCl_2_IN_2_O: C, 34.36; H, 2.88; N, 4.01. Found: C, 34.68; H, 2.85; N, 4.04.

**6g**: yield: 83%; mp 177 °C; *υ*_max_(ATR)/cm^−1^ 3064, 2972, 2931, 2833, 1628, 1605, 1579, 1544, 1512, 1460, 1438, 1409, 1377, 1344, 1306, 1292, 1250, 1175, 1102, 1050, 1026, 971, 888, 845, 806, 752, 734, 682; ^1^H NMR (500 MHz, CDCl_3_) δ 7.68 (t, *J* = 1.8 Hz, 1H, *H*^ar^), 7.31 (d, *J* = 1.7 Hz, 2H, *H*^ar^), 7.12 (d, *J* = 8.7 Hz, 2H, *H*^ar^), 6.91 (d, *J* = 8.7 Hz, 2H, *H*^ar^), 4.17 (vdq, *J* = 17 Hz, 7.1 Hz, 4H, NC*H*_2_), 3.83 (s, 3H, OC*H*_3_), 1.32 (vdt, *J* = 21 Hz, 7.2 Hz, 6H, CH_2_C*H*_3_); ^13^C NMR (125 MHz, CDCl_3_) δ 180.7 (N*C*N), 160.7 (C^ar^), 135.2 (C^ar^), 132.2 (C^ar^), 132.0 (C^ar^), 131.9 (C^ar^), 131.5 (C^ar^), 127.9 (C^ar^), 123.4 (C^ar^), 118.7 (C^ar^), 114.7 (C=C), 55.50 (O*C*H_3_), 44.36 (N*C*H_2_), 44.18 (N*C*H_2_), 17.20 (CH_2_*C*H_3_), 17.07 (CH_2_*C*H_3_); *m*/*z* (%) 790 (23) [M^+^], 788 (52) [M^+^], 786 (25) [M^+^], 663 (66) [M^+^–I], 661 (100) [M^+^–I], 659 (54) [M^+^–I]; anal. calcd. for C_20_H_20_AuBr_2_IN_2_O: C, 30.48; H, 2.56; N, 3.55. Found: C, 30.81; H, 2.52; N, 3.58.

**6h**: yield: 63%; mp 181–182 °C; *υ*_max_(ATR)/cm^−1^ 2989, 2950, 2931, 2900, 1610, 1598, 1582, 1520, 1506, 1460, 1417, 1379, 1362, 1346, 1309, 1294, 1252, 1237, 1174, 1136, 1090, 1022, 975, 889, 877, 833, 819, 808, 797, 778, 762, 736, 718, 687, 666; ^1^H NMR (300 MHz, CDCl_3_) δ 7.11 (d, *J* = 8.8 Hz, 2H, *H*^ar^), 6.9–6.7 (m, 4H, *H*^ar^), 6.62 (s, 1H, *H*^ar^), 4.2–4.1 (m, 4H, NC*H*_2_), 3.85 (s, 3H, OC*H*_3_), 3.77 (s, 3H, OC*H*_3_), 3.72 (s, 3H, OC*H*_3_), 1.4–1.2 (m, 6H, CH_2_C*H*_3_); ^13^C NMR (75.5 MHz, CDCl_3_) δ 179.3 (N*C*N), 160.1 (C^ar^), 149.6 (C^ar^), 148.9 (C^ar^), 131.7 (C^ar^), 130.7 (C^ar^), 130.5 (C^ar^), 123.2 (C^ar^), 120.1 (C^ar^), 119.8 (C^ar^), 114.3 (C^ar^), 113.3 (C^ar^), 111.1 (C^ar^), 55.9 (O*C*H_3_), 55.8 (O*C*H_3_), 55.3 (O*C*H_3_), 44.0 (N*C*H_2_), 43.9 (N*C*H_2_), 17.1 (CH_2_*C*H_3_), 16.9 (CH_2_*C*H_3_); *m*/*z* (%) 690 (27) [M^+^], 660 (7), 564 (100) [M^+^–I], 547 (13), 533 (17), 282 (8); anal. calcd. for C_22_H_26_AuIN_2_O_3_: C, 38.28; H, 3.80; N, 4.06. Found: C, 38.72; H, 3.83; N, 4.03.

**6i**: yield: 79%; mp 168–169 °C; *υ*_max_(ATR)/cm^−1^ 2971, 2938, 2838, 1602, 1574, 1520, 1505, 1459, 1442, 1416, 1371, 1345, 1316, 1302, 1290, 1269, 1245, 1227, 1175, 1133, 1121, 1109, 1089, 1047, 1025, 1009, 977, 961, 898, 887, 837, 807, 777, 762, 736, 720, 687, 666; ^1^H NMR (300 MHz, CDCl_3_) δ 7.09 (d, *J* = 8.5 Hz, 2H, *H*^ar^), 6.9–6.8 (m, 5H, *H*^ar^), 4.2–4.1 (m, 4H, NC*H*_2_), 3.86 (s, 3H, OC*H*_3_), 3.78 (s, 3H, OC*H*_3_), 1.4–1.2 (m, 6H, CH_2_C*H*_3_); ^13^C NMR (75.5 MHz, CDCl_3_) δ 179.6 (N*C*N), 160.3 (C^ar^), 153.5–150.2 (m, C^ar^), 148.5 (C^ar^), 131.8–131.7 (m, C^ar^), 131.0 (C^ar^), 129.4 (C^ar^), 126.9 (C^ar^), 120.3–120.2 (m, C^ar^), 119.4 (C^ar^), 118.3–118.0 (m, (C^ar^)), 114.4–114.3 (m, C^ar^), 113.4 (C^ar^), 56.1 (O*C*H_3_), 55.3 (O*C*H_3_), 44.0 (N*C*H_2_), 43.9 (N*C*H_2_), 16.9 (CH_2_*C*H_3_); *m*/*z* (%) 678 (48) [M^+^], 660 (7), 552 (100) [M^+^–I], 276 (12); anal. calcd. for C_21_H_23_AuFIN_2_O_2_: C, 37.19; H, 3.42; N, 4.13. Found: C, 37.74; H, 3.34; N, 4.28.

**6j**: yield: 74%; *υ*_max_(ATR)/cm^−1^ 2969, 2931, 2835, 1608, 1593, 1573, 1516, 1494, 1410, 1378, 1345, 1288, 1249, 1175, 1147, 1109, 1089, 1050, 1020, 968, 888, 847, 831, 808, 722, 681; ^1^H NMR (300 MHz, CDCl_3_) δ 7.40 (s, 1H, *H*^ar^), 7.1–7.0 (m, 3H, *H*^ar^), 6.9–6.8 (m, 3H, *H*^ar^), 4.2–4.1 (m, 4H, NC*H*_2_), 3.87 (s, 3H, OC*H*_3_), 3.78 (s, 3H, OC*H*_3_), 1.4–1.2 (m, 6H, CH_2_C*H*_3_); ^13^C NMR (75.5 MHz, CDCl_3_) δ 179.6 (N*C*N), 160.3 (C^ar^), 156.5 (C^ar^), 135.0 (C^ar^), 131.7 (C^ar^), 131.1 (C^ar^), 131.0 (C^ar^), 129.1 (C^ar^), 121.2 (C^ar^), 119.8 (C^ar^), 119.4 (C^ar^), 114.4 (C^ar^), 114.3 (C^ar^), 111.9 (C^ar^), 56.3 (O*C*H_3_), 55.3 (O*C*H_3_), 44.0 (N*C*H_2_), 43.9 (N*C*H_2_), 17.0 (CH_2_*C*H_3_), 16.9 (CH_2_*C*H_3_); *m*/*z* (%) 740 (59) [M^+^], 738 (61) [M^+^], 660 (76), 613 (73) [M^+^–I], 611 (71) [M^+^–I], 533 (100); anal. calcd. for C_21_H_23_AuBrIN_2_O_2_: C, 34.12; H, 3.14; N, 3.79. Found: C, 34.47; H, 3.20; N, 3.85.

**6k**: mp 207–208 °C; *υ*_max_(ATR)/cm^−1^ 3000, 2959, 2934, 2868, 2835, 1607, 1579, 1515, 1502, 1459, 1412, 1379, 1347, 1306, 1295, 1240, 1177, 1125, 1050, 1030, 1004, 976, 885, 859, 840, 827, 811, 785, 763, 736, 692, 670; ^1^H NMR (300 MHz, CDCl_3_) δ 7.17 (d, *J* = 8.8 Hz, 2H, *H*^ar^), 6.92 (d, *J* = 8.8 Hz, 2H, *H*^ar^), 6.40 (s, 2H, *H*^ar^), 4.3–4.1 (m, 4H, NC*H*_2_), 3.87 (s, 3H, OC*H*_3_), 3.84 (s, 3H, OC*H*_3_), 3.80 (s, 6H, OC*H*_3_), 1.5–1.3 (m, 6H, CH_2_C*H*_3_); ^13^C NMR (75.5 MHz, CDCl_3_) δ 179.7 (N*C*N), 160.3 (C^ar^), 153.3 (C^ar^), 138.8 (C^ar^), 131.8 (C^ar^), 130.8 (C^ar^), 130.6 (C^ar^), 123.0 (C^ar^), 119.8 (C^ar^), 114.3 (C^ar^), 107.8 (C^ar^), 60.9 (O*C*H_3_), 56.4 (O*C*H_3_), 56.2 (O*C*H_3_), 55.3 (O*C*H_3_), 44.1 (N*C*H_2_), 43.9 (N*C*H_2_), 17.2 (CH_2_*C*H_3_), 17.0 (CH_2_*C*H_3_); *m*/*z* (%) 720 (46) [M^+^], 594 (100) [M^+^–I]; anal. calcd. for C_23_H_28_AuIN_2_O_4_: C, 38.35; H, 3.92; N, 3.89. Found: C, 38.63; H, 3.88; N, 3.94.

**6l**: yield: 58%; mp 192–193 °C; *υ*_max_(ATR)/cm^−1^ 2972, 2935, 2833, 1607, 1592, 1563, 1515, 1492, 1460, 1411, 1317, 1294, 1270, 1251, 1175, 1157, 1123, 1108, 1091, 1075, 1041, 1001, 976, 903, 870, 834, 811, 787, 755, 708, 687, 665; ^1^H NMR (300 MHz, CDCl_3_) δ 7.12 (d, *J* = 8.7 Hz, 2H, *H*^ar^), 6.88 (d, *J* = 8.7 Hz, 2H, *H*^ar^), 6.85 (s, 1H, *H*^ar^), 6.56 (s, 1H, *H*^ar^), 4.2–4.1 (m, 4H, NC*H*_2_), 3.85 (s, 3H, OC*H*_3_), 3.80 (s, 3H, OC*H*_3_), 3.70 (s, 3H, OC*H*_3_), 1.35 (t, *J* = 7.2 Hz, 3H, CH_2_C*H*_3_), 1.29 (t, *J* = 7.2 Hz, 3H, CH_2_C*H*_3_); ^13^C NMR (75.5 MHz, CDCl_3_) δ 179.9 (N*C*N), 160.4 (C^ar^), 153.8 (C^ar^), 146.2 (C^ar^), 131.8 (C^ar^), 131.1 (C^ar^), 129.4 (C^ar^), 128.7 (C^ar^), 124.0 (C^ar^), 123.8 (C^ar^), 119.3 (C^ar^), 114.4 (C^ar^), 114.3 (C^ar^), 113.2 (C^ar^), 60.8 (O*C*H_3_), 56.2 (O*C*H_3_), 55.3 (O*C*H_3_), 44.0 (N*C*H_2_), 43.9 (N*C*H_2_), 17.2 (CH_2_*C*H_3_), 16.9 (CH_2_*C*H_3_); *m*/*z* (%) 726 (20) [M^+^], 724 (48) [M^+^], 660 (13), 598 (100) [M^+^–I], 534 (23), 142 (16), 127 (8); anal. calcd. for C_22_H_25_AuClIN_2_O_3_: C, 36.46; H, 3.48; N, 3.87. Found: C, 36.70; H, 3.43; N, 3.84.

**6m**: yield: 73%; mp 194–195 °C; *υ*_max_(ATR)/cm^−1^ 2967, 2941, 2835, 1607, 1588, 1558, 1514, 1489, 1460, 1410, 1344, 1318, 1291, 1266, 1250, 1234, 1179, 1156, 1110, 1091, 1075, 1037, 1024, 991, 899, 862, 843, 813, 789, 753, 738, 698, 665; ^1^H NMR (300 MHz, CDCl_3_) δ 7.12 (d, *J* = 8.8 Hz, 2H, *H*^ar^), 7.02 (s, 1H, *H*^ar^), 6.88 (d, *J* = 8.8 Hz, 2H, *H*^ar^), 6.60 (s, 1H, *H*^ar^), 4.2–4.1 (m, 4H, NC*H*_2_), 3.84 (s, 3H, OC*H*_3_), 3.80 (s, 3H, OC*H*_3_), 3.69 (s, 3H, OC*H*_3_), 1.35 (t, *J* = 7.2 Hz, 3H, CH_2_C*H*_3_), 1.23 (t, *J* = 7.3 Hz, 3H, CH_2_C*H*_3_); ^13^C NMR (75.5 MHz, CDCl_3_) δ 179.8 (NCN), 160.4 (C^ar^), 153.6 (C^ar^), 147.2 (C^ar^), 131.8 (C^ar^), 131.1 (C^ar^), 129.2 (C^ar^), 126.6 (C^ar^), 124.6 (C^ar^), 119.3 (C^ar^), 117.9 (C^ar^), 114.4 (C^ar^), 113.9 (C^ar^), 60.7 (OCH_3_), 56.1 (O*C*H_3_), 55.3 (O*C*H_3_), 44.1 (N*C*H_2_), 44.0 (N*C*H_2_), 17.2 (CH_2_*C*H_3_), 16.9 (CH_2_*C*H_3_); *m*/*z* (%) 770 (48) [M^+^], 768 (46) [M^+^], 725 (21), 690 (22), 643 (100) [M^+^–I], 641 (99) [M^+^–I], 563 (42), 142 (93), 127 (40), 94 (27), 43 (65); anal. calcd. for C_22_H_25_AuBrIN_2_O_3_: C, 34.35; H, 3.28; N, 3.64. Found: C, 34.70; H, 3.33; N, 3.60.

**6n**: yield: 71%; mp 118–120 °C; *υ*_max_(ATR)/cm^−1^ 2966, 2932, 2834, 1606, 1584, 1550, 1513, 1482, 1460, 1409, 1344, 1316, 1292, 1250, 1176, 1151, 1110, 1091, 1075, 1029, 995, 895, 863, 838, 800, 751, 689, 658; ^1^H NMR (300 MHz, CDCl_3_) δ 7.24 (s, 1H, *H*^ar^), 7.12 (d, *J* = 8.8 Hz, 2H, *H*^ar^), 6.88 (d, *J* = 8.8 Hz, 2H, *H*^ar^), 6.62 (s, 1H, *H*^ar^), 4.2–4.1 (m, 4H, NC*H*_2_), 3.81 (s, 3H, OC*H*_3_), 3.80 (s, 3H, OC*H*_3_), 3.67 (s, 3H, OC*H*_3_), 1.35 (t, *J* = 7.2 Hz, 3H, CH_2_C*H*_3_), 1.28 (t, *J* = 7.2 Hz, 3H, CH_2_CH_3_); ^13^C NMR (75.5 MHz, CDCl_3_) δ 179.8 (NCN), 160.3 (C^ar^), 152.3 (C^ar^), 149.6 (C^ar^), 132.2 (C^ar^), 131.8 (C^ar^), 131.0 (C^ar^), 129.0 (C^ar^), 125.5 (C^ar^), 119.3 (C^ar^), 115.0 (C^ar^), 114.4 (C^ar^), 114.3 (C^ar^), 92.5 (C^ar^), 60.5 (O*C*H_3_), 56.0 (O*C*H_3_), 55.3 (O*C*H_3_), 44.1 (N*C*H_2_), 43.9 (N*C*H_2_), 17.2 (CH_2_*C*H_3_), 16.9 (CH_2_*C*H_3_); *m*/*z* (%) 816 (52) [M^+^], 689 (87) [M^+^–I], 660 (23), 564 (12), 533 (100), 345 (13), 142 (75), 127 (27); anal. calcd. for C_22_H_25_AuI_2_N_2_O_3_: C, 32.37; H, 3.09; N, 3.43. Found: C, 32.58; H, 3.16; N, 3.38.

**6o**: yield: 44%; mp 178–179 °C; *υ*_max_(ATR)/cm^−1^ 3005, 2963, 2938, 2831, 1607, 1579, 1517, 1502, 1455, 1404, 1318, 1293, 1236, 1173, 1123, 1018, 998, 913, 843, 814, 790, 766, 736, 683, 665; ^1^H NMR (300 MHz, CDCl_3_) δ 7.11 (d, *J* = 8.8 Hz, 2H, *H*^ar^), 6.87 (d, *J* = 8.8 Hz, 2H, *H*^ar^), 6.36 (s, 2H, *H*^ar^), 3.83 (s, 3H, OC*H*_3_), 3.79 (s, 3H, OC*H*_3_), 3.76 (s, 3H, OC*H*_3_), 3.73 (s, 3H, OC*H*_3_), 3.71 (s, 3H, NC*H*_3_), 3.70 (s, 3H, NC*H*_3_); ^13^C NMR (75.5 MHz, CDCl_3_) δ 180.8 (N*C*N), 160.3 (C^ar^), 153.3 (C^ar^), 138.8 (C^ar^), 131.7 (C^ar^), 131.3 (C^ar^), 122.8 (C^ar^), 119.6 (C^ar^), 114.4 (C^ar^), 110.0 (C^ar^), 107.7 (C^ar^), 60.9 (O*C*H_3_), 56.2 (O*C*H_3_), 55.3 (O*C*H_3_), 36.4 (N*C*H_3_), 36.3 (N*C*H_3_); *m*/*z* (%) 692 (47) [M^+^], 566 (100) [M^+^–I], 355 (28), 339 (23), 283 (19), 142 (63), 127 (27); anal. calcd. for C_21_H_24_AuIN_2_O_4_: C, 36.43; H, 3.49; N, 4.05. Found: C, 36.65; H, 3.43; N, 4.10.

### 4.4. Crystal Structure Analysis of **6c**

X-ray structure analysis of single crystals of the complex **6c** was performed on a Stoe StadiVari diffractometer equipped with a graphite-monochromated Mo-*K*_α_ (*λ* = 0.71073 Å) radiation source and an Oxford Cryostream low-temperature unit. A suitable single crystal of **6c** was embedded in inert perfluorinated oil (Fomblin^®^ YR-1800) and mounted on a nylon loop before collecting data at 170(2) K.

Data were corrected for Lorentz and polarization effects; a spherical absorption correction was applied. The structures were solved by direct method SHELXT 2014/5 and refined by full-matrix least-squares procedures on *F*_0_^2^ − *F*_c_^2^ with SHELXL 2018/3, interfaced by WinGX [55,56,57]. All non-hydrogen atoms were refined with anisotropic displacement parameters. Hydrogen atoms were calculated in idealized positions with fixed displacement parameters during refinement. Occupational disorder of bromine and the phenyl-appended methoxy group was resolved in one of the two symmetry-independent units of **6c**. Mercury was used for structure illustrations/graphical output [58].

### 4.5. Anticancer Activity

#### 4.5.1. Cell Line and Culture Conditions

EAC cells FLO-1 and SK-GT-4 (a gift from Dr. Shrikant Anant’s lab, University of Kansas Medical Center, Kansas City), were cultured in complete DMEM (4.5 g/L glucose, sodium pyruvate and L-glutamine, Corning, MA). The complete DMEM was prepared by adding fetal bovine serum (10% FBS, heat-inactivated, Sigma-Aldrich, MO) and 1% antibiotic-antimycotic solution (Corning, MA). EAC cells were cultured in 5% CO_2_ at 37 °C. All procedures were performed according to the standard guidelines and regulations and as per the manufacturers’ instructions.

#### 4.5.2. Proliferation Assay

A total of 5000 EAC cells/well (SK-GT-4 and FLO-1) were plated in a 96-well plate using complete DMEM. After 24 h of plating, EAC cells were treated with gold complexes at different concentrations. After 72 h, the medium was removed, and cell viability was measured using the hexosaminidase enzymatic assay [59]. The percentage of inhibition was calculated by comparing cell viability after compound treatment with controls.

#### 4.5.3. Colony Formation Assay

A total of 500 cells/well of EAC were plated in 6-well plates. After 24 h, the EAC cells were treated with IC_50_ and semi-IC_50_ concentrations of **6b**, **6d,** and **6i**. Compounds containing media were replaced after 72 h with complete DMEM to remove the test compounds. The cells were grown for 10–12 days to form colonies. The resulting colonies were washed and fixed using a 10% formalin solution. After 20 min, the formalin was removed, and the fixed cells were washed and stained with 1% crystal violet solution in 10% ethanol. After staining, colonies were washed to remove crystal violet, dried, counted, and compared to controls [60]. We scanned the stained and dried 6-well plates using a Canon Image RUNNER Advance scanner to make figures.

#### 4.5.4. Cell Cycle Analysis

A total of 200,000 EAC cells (SK-GT-4 and FLO-1) per well were plated in 6-well plates. After 24 h, EAC cells were treated with IC_50_ and semi-IC_50_ concentrations of compounds **6b**, **6d,** and **6i**. After 72 h, EAC cells were washed, resuspended in PBS, and fixed using an ice-cold fixing solution (70% ethanol in PBS), followed by storage overnight at 4 °C. The next day, EAC cells were centrifuged, washed with PBS, resuspended, permeabilized, and stained with FxCycleTM PI/RNase staining solution (Invitrogen). The cell cycle was studied by flow cytometry using an FACS Calibur analyzer (Becton Dickinson, Mountain View, CA, USA). The experimental datasets were plotted using ModFit LT™ software (Verity Software House, Topsham, ME, USA).

#### 4.5.5. Apoptosis Assay

A total of 200,000 EAC cells/well were plated in a 6-well plate in complete DMEM, and, after 24 h, the EAC cells were treated with IC_50_ concentrations of compounds **6b**, **6d,** and **6i**. After 72 h, cells were trypsinized, washed and stained using the Annexin V-FITC Early Apoptosis Detection Kit (Cell Signaling Technology#6592) following the manufacturer’s instructions, and studied by flow cytometry.

#### 4.5.6. Spheroid Formation Assay

A total of 500 EAC cells were plated in an ultra-low attachment 96-well plate (96-well, Corning, Lowell, MA, USA) in spheroid medium prepared from serum-free DMEM supplemented with heparin salt (4 µg/mL), EGF (20 ng/mL), FGF (20 ng/mL), 1% antibiotic-antimycotic, and B27 supplement. After 2 days, spheroids were treated with IC_50_ and semi-IC_50_ concentrations of **6b**, **6d,** and **6i**. After 7 days, spheroids were counted and imaged [60].

#### 4.5.7. Western Blot Analysis

A total of 500,000 cells of EAC cell lines were plated in a 10 cm cell culture Petri dish, and, after 24 h, cells were incubated with IC_50_ concentrations of **6b**, **6d,** and **6i** for 72 h. Cells were washed and lysed in lysis buffer with phosphatase and protease inhibitor (Roche), followed by sonification. The protein lysate was centrifuged at 6000 rpm for 10 min at 4 °C. Protein determination was performed using the Pierce BCA protein assay kit to estimate protein contents. A total of 50 µg of protein from each group was subjected to gel electrophoresis and further transferred onto polyvinylidene difluoride membranes (Millipore, Bedford, MA, USA) at 90 V for 2 h under cold conditions. These PVDF membranes were then blocked for 1 h by using 5% milk in TBST, washed with TBST, and incubated with primary antibodies at 4 °C overnight. The next day, membranes were washed using TBST to remove primary antibodies and incubated with respective secondary anti-mouse and anti-rabbit antibodies (Cell Signaling Technology, anti-mouse#7076, anti-rabbit#7074) for 1 h. The proteins were identified by using the GE Health Care chemiluminescence system (Piscataway, NJ, USA), imaged using the Bio-Rad ChemiDoc-XRS+ instrument, and processed by image lab. Antibodies for detecting cyclin D1 (CST#2922), Bcl-XL (CST#2762), Bcl-2 (CST#4223), c-Myc (CST#9402), Bax (CST#2772), Mcl-1 (CST#4572), and PARP (CST#9542) were bought from Cell Signaling Technology (Beverly, MA, USA), and GAPDH (G-9) was purchased from Santa Cruz Biotech Inc. (Santa Cruz, CA, USA).

#### 4.5.8. Statistical Analysis

All values are shown as the mean ± SD. Experimental data were examined using an unpaired two-tailed *t*-test by comparing to the corresponding control group. A probability value of less than 0.05 was considered as statistically significant (* *p* < 0.05, ** *p* < 0.01).

## 5. Conclusions

The NHC ligand system of a previously published anticancer active iodidogold(I)–NHC complex was successfully optimized in terms of anticancer properties, and high activities against EAC cells were achieved for several new gold complexes. These compounds induced programmed cell death and suppressed colony and spheroid formation by EAC cells at low doses. Together with their promising suppressive effects on cyclin D1 and *c*-Myc expression, there exists a considerable potential of iodidogold(I)–NHC complexes as new candidates for the treatment of problematic tumor diseases such as EAC. Deeper investigations of the mechanisms of action of these gold compounds will provide more information about their prospects as new anticancer drugs. An extension of compound testing to other tumor entities than EAC will also be of great interest given the described anticancer properties of the newly discovered gold complexes.

## Figures and Tables

**Table 1:** Crystallographic data of the iodidogold complex **6c**.

	6c
CCDC number	2235874
Sum formula	C20.16 H21.47 Au Br0.84 I N2 O1.16
*M*/gmol^−1^	701.56
Crystal system	monoclinic
Space group	*P2_1_/n* (Nr. 14)
Crystal description	Pale yellow block
Crystal size/mm	0.24 × 0.16 × 0.13
*a/*Å	9.8361(3)
*b/*Å	13.1351(6)
*c/*Å	16.7031(5)
*α*/°	90
*β*/°	101.576(2)
*γ*/°	90
*V/*Å^3^	2114.11(13)
Z	4
*ρ*_calculated/_gcm^−3^	2.204
*µ*/mm^−1^	10.020
*θ*_range_/°	2.7–28.7
Radiation	Mo-K_α_ (0.71073 Å)
*T/*K	170(2)
Measured reflections	16653
Independent reflections	5028
Reflections with *I* > 2 *s*(*I*)	3475
*R*_int_	0.078
Restraints and parameters	18 and 268
*R*_1_ [*F*^2^ > 2 *s* (*F*^2^)]	0.0440
w*R*_2_(*F*^2^)	0.0980
GooF (*S*)	0.980
*ρ*_residual_ (largest peak and hole)/eÅ^−3^	0.920, −1.884

**Table 2:** Inhibitory activities (IC_50_ values) of compounds **5a–g** and **6a–o**. Complex **1b** served as positive control. IC_50_ values are presented in µM concentrations at the 72 h time-point.

Compound	SK-GT-4	FLO-1
**1b**	1.31 ± 0.39	0.95 ± 0.15
**5a**	3.9 ± 0.71	1.5 ± 0.17
**5b**	>40	29.8 ± 0.52
**5c**	>40	27.33 ± 0.83
**5d**	>40	29.2 ± 0.52
**5e**	>40	28.13 ± 0.83
**5f**	>40	4.5 ± 0.28
**5g**	23.2 ± 0.92	4.78 ± 0.35
**6a**	15.33 ± 8.96	0.51 ± 0.01
**6b**	0.26 ± 0.09	0.12 ± 0.01
**6c**	>10	0.5 ± 0.02
**6d**	1.1 ± 0.06	0.58 ± 0.07
**6e**	>10	1.0 ± 0.06
**6f**	3.78 ± 1.25	0.9 ± 0.04
**6g**	23.53 ± 7.71	1.4 ± 0.10
**6h**	7.4 ± 0.50	0.55 ± 0.03
**6i**	0.325 ± 0.06	0.3 ± 0.02
**6j**	1.2 ± 0.08	0.625 ± 0.06
**6k**	13.4 ± 4.10	4.07 ± 2.21
**6l**	>10	>10
**6m**	0.4 ± 0.04	0.45 ± 0.09
**6n**	9.2 ± 1.71	0.95 ± 0.03
**6o**	2.9 ± 0.26	1.43 ± 0.25

## Data Availability

Original data can be requested from the corresponding author.

## References

[1] Sung H., Ferlay J., Siegel R.L., Laversanne M., Soerjomataram I., Jemal A., Bray F. (2021). Global Cancer Statistics 2020: GLOBOCAN Estimates of Incidence and Mortality Worldwide for 36 Cancers in 185 Countries. CA Cancer J. Clin..

[2] Uhlenhopp D.J., Then E.O., Sunkara T., Gaduputi V. (2020). Epidemiology of esophageal cancer: Update in global trends, etiology and risk factors. Clin. J. Gastroenterol..

[3] Njeij B., McCarty T.R., Birk J.W. (2016). Trends in esophageal cancer survival in United States adults from 1973 to 2009: A SEER database analysis. J. Gastroenterol. Hepatol..

[4] Siegel R.L., Miller K.D., Fuchs H.E., Jemal A. (2021). Cancer Statistics, 2021. CA Cancer J. Clin..

[5] Ratajczak T., Kumar G., Albandar H., Redlien P., Yacyshyn S., Markert R. (2016). Metabolic characteristics of esophageal carcinoma in a veteran population. J. Clin. Oncol..

[6] Xu Q.L., Li H., Zhu Y.J., Xu G. (2020). The treatments and postoperative complications of esophageal cancer: A review. J. Cardiothorac. Surg..

[7] Patel N., Benipal B. (2018). Incidence of Esophageal Cancer in the United States from 2001–2015: A United States Cancer Statistics Analysis of 50 States. Cureus.

[8] Blot W.J., Tarone R.E., Thun M., Linet M.S., Cerhan J.R., Haiman C.A., Schottenfeld D. (2017). Esophageal Cancer. Cancer Epidemiology and Prevention.

[9] Wang H., Li S., Liu T., Chen J., Dang J. (2022). Neoadjuvant immune checkpoint inhibitor in combination with chemotherapy or chemoradiotherapy in resectable esophageal cancer: A systematic review and meta-analysis. Front. Immunol..

[10] Shaw C.F. (1999). Gold-based therapeutic agents. Chem. Rev..

[11] Harris G.J., Turner J.N., Von Hoff D.D. (1986). Growth of carcinoma of the esophagus and gastroesophageal junction in a human tumor cloning assay. Cancer Drug Deliv..

[12] Onodera T., Momose I., Kawada M. (2019). Potential anticancer activity of auranofin. Chem. Pharm. Bull..

[13] Liu N., Li X., Huang H., Zhao C., Liao S., Yang C., Liu S., Song W., Lu X., Lan X. (2014). Clinically used antirheumatic agent auranofin is a proteasomal deubiquitinase inhibitor and inhibits tumor growth. Oncotarget.

[14] Huang H., Liao Y., Liu N., Hua X., Cai J., Yang C., Long H., Zhao C., Chen X., Lan X. (2016). Two clinical drugs deubiquitinase inhibitor auranofin and aldehyde dehydrogenase inhibitor disulfiram trigger synergistic anti-tumor effects in vitro and in vivo. Oncotarget.

[15] Thangamani S., Maland M., Mohammad H., Pascuzzi P.E., Avramova L., Koehler C.M., Hazbun T.R., Seleem M.N. (2017). Repurposing approach identifies auranofin with broad spectrum antifungal activity that targets Mia40-Erv1 pathway. Front. Cell. Infect. Microbiol..

[16] Dickson-Murray E., Nedara K., Modjtahedi N., Tokatlidis K. (2021). The Mia40/CHCHD4 oxidative folding system: Redox regulation and signaling in the mitochondrial intermembrane space. Antioxidants.

[17] Landini I., Massai L., Cirri D., Gamberi T., Paoli P., Messori L., Mini E., Nobili S. (2020). Structure-activity relationships in a series of auranofin analogues showing remarkable antiproliferative properties. J. Inorg. Biochem..

[18] Massai L., Cirri D., Marzo T., Messori L. (2022). Auranofin and its analogs as prospective agents for the treatment of colorectal cancer. Cancer Drug Resist..

[19] Kober L., Schleser S.W., Bär S.I., Schobert R. (2022). Revisiting the anticancer properties of phosphane(9-ribosylpurine-6-thiolato)gold(I) complexes and their 9H-purine precursors. J. Biol. Inorg. Chem..

[20] Schmidt C., Karge B., Misgeld R., Prokop A., Brönstrup M., Ott I. (2017). Biscarbene gold(I) complexes: Structure-activity-relationships regarding antibacterial effects, cytotoxicity, TrxR inhibition and cellular bioavailability. Med. Chem. Commun..

[21] Schmidt C., Albrecht L., Balasupramaniam S., Misgeld R., Karge B., Brönstrup M., Prokop A., Baumann K., Reichl S., Ott I. (2019). A gold(I) biscarbene complex with improved activity as a TrxR inhibitor and cytotoxic drug: Comparative studies with different gold metallodrugs. Metallomics.

[22] Schuh E., Pflüger C., Citta A., Folda A., Rigobello M.P., Bindoli A., Casini A., Mohr F. (2012). Gold(I) carbene complexes causing thioredoxin 1 and thioredoxin 2 oxidation as potential anticancer agents. J. Med. Chem..

[23] Wragg D., de Almeida A., Bonsignore R., Kühn F.E., Leoni S., Casini A. (2018). On the mechanism of gold/BHC compounds binding to DNA G-quadruplexes: Combined metadynamics and biophysical methods. Angew. Chem. Int. Ed..

[24] Tialiou A., Chin J., Keppler B.K., Reithofer M.R. (2022). Current developments of *N*-heterocyclic carbene (Au(I)/Au(III)) complexes toward cancer treatment. Biomedicines.

[25] Kaps L., Biersack B., Müller-Bunz H., Mahal K., Münzner J., Tacke M., Mueller T., Schobert R. (2012). Gold(I)-NHC complexes of antitumoral diarylimidazoles: Structures, cellular uptake routes and anticancer activities. J. Inorg. Biochem..

[26] Münzner J., Biersack B., Kaps L., Schobert R., Sasse F. (2013). Synergistic “gold effects” of anti-vascular 4,5-diarylimidazol-2-ylidene gold(I) carbene complexes. Int. J. Clin. Pharmacol. Ther..

[27] Muenzner J.K., Biersack B., Kalie H., Andronache I.C., Kaps L., Schuppan D., Sasse F., Schobert R. (2014). Gold(I) biscarbene complexes derived from vascular-disrupting combretastatin A-4 address different targets and show antimetastatic potential. ChemMedChem.

[28] Hatem E., El Banna N., Heneman-Masurel A., Baille D., Vernis L., Riquier S., Golinelli-Cohen M.-P., Guittet O., Vallières C., Camadro J.-M. (2022). Novel insights into redox-based mechanisms for auranofin-induced rapid cancer cell death. Cancers.

[29] Bian M., Fan R., Jiang G., Wang Y., Lu Y., Liu W. (2020). Halo and pseudohalo gold(I)-NHC complexes derived from 4,5-diarylimidazoles with excellent in vitro and in vivo anticancer activities against HCC. J. Med. Chem..

[30] Plante J.P., Glass T.E. (2006). Shape-selective fluorescent sensing ensemble using a tweezer-type metalloreceptor. Org. Lett..

[31] Benitez D., Shapiro N.D., Tkatchouk E., Wang Y., Goddard III W.A., Toste F.D. (2009). A bonding model for gold(I) carbene complexes. Nat. Chem..

[32] Das P.K., Islam F., Smith R.A., Lam A.K. (2021). Therapeutic strategies against cancer stem cells in esophageal carcinomas. Front. Oncol..

[33] Liao D.J., Thakur A., Wu J., Biliran H., Sarkar F.H. (2007). Perspectives on c-Myc, Cyclin D1, and their interaction in cancer formation, progression, and response to chemotherapy. Crit. Rev. Oncog..

[34] Muenzner J.K., Biersack B., Albrecht A., Rehm T., Lacher U., Milius W., Casini A., Zhang J.-J., Ott I., Brabec V. (2016). Ferrocenyl-coupled *N*-heterocyclic carbene complexes of gold(I): A successful approach to multinuclear anticancer drugs. Chem. Eur. J..

[35] Bär S.I., Gold M., Schleser S.W., Rehm T., Bär A., Köhler L., Carnell L.R., Biersack B., Schobert R. (2021). Guided antitumoural drugs: (imidazol-2-ylidene)(L)gold(I) complexes seeking cellular targets controlled by the nature of ligand L. Chem. Eur. J..

[36] Paloque L., Hemmert C., Valentin A., Gornitzka H. (2015). Synthesis, characterization, and antileishmanial activities of gold(I) complexes involving quinoline functionalized *N*-heterocyclic carbenes. Eur. J. Med. Chem..

[37] Tong Z., Chatterjee D., Deng D., Veeranki O., Mejia A., Ajani J.A., Hofstetter W., Lin S., Guha S., Kopetz S. (2017). Antitumor effects of cyclin dependent kinase 9 inhibition in esophageal adenocarcinoma. Oncotarget.

[38] Su W., Guo C., Wang L., Wang Z., Yang X., Niu F., Tzou D., Yang X., Huang X., Wu J. (2019). LncRNA *MIR22HG* abrogation inhibits proliferation and induces apoptosis in esophageal adenocarcinoma cells via activation of the STAT3/*c*-Myc/FAK signaling. Aging.

[39] Vangamudi B., Zhu S., Soutto M., Belkhiri A., El-Rifai W. (2011). Regulation of β-catenin by t-DARPP in upper gastrointestinal cancer cells. Mol. Cancer.

[40] Madden S.K., De Araujo A.D., Gerhardt M., Fairlie D.P., Mason J.M. (2021). Taking the Myc out of cancer: Toward therapeutic strategies to directly inhibit *c*-Myc. Mol. Cancer.

[41] Montalto F.I., De Amicis F. (2020). Cyclin D1 in cancer: A molecular connection for cell cycle control, adhesion and invasion in tumor and stroma. Cells.

[42] Taylor C., Loomans H.A., Le Bras G.F., Koumangoye R.B., Romero-Morales A.I., Quast L.L., Zaika A.I., El-Rifai W., Andl T., Andl C.D. (2015). Activin a signaling regulates cell invasion and proliferation in esophageal adenocarcinoma. Oncotarget.

[43] Chen C., Ma Z., Jiang H. (2021). EMT participates in the regulation of exosomes secretion and function in esophageal cancer cells. Technol. Cancer Res. Treat..

[44] Silvers A.L., Lin L., Bass A.J., Chen G., Wang Z., Thomas D.G., Lin J., Giordano T.J., Orringer M.B., Beer D.G. (2010). Decreased selenium-binding protein 1 in esophageal adenocarcinoma results from posttranscriptional and epigenetic regulation and affects chemosensitivity. Clin. Cancer Res..

[45] Ahrens T.D., Timme S., Ostendorp J., Bogatyreva L., Hoeppner J., Hopt U.T., Hauschke D., Werner M., Lassmann S. (2016). Response of esophageal cancer cells to epigenetic inhibitors is mediated via altered thioredoxin activity. Lab. Investig..

[46] Feingold P.L., Surman D.R., Brown K., Xu Y., McDuffie L.A., Shukla V., Reardon E.S., Crooks D.R., Trepel J.B., Lee S. (2018). Induction of thioredoxin-interacting protein by a histone deacetylase inhibitor, entinostat, is associated with DNA damage and apoptosis in esophageal adenocarcinoma. Mol. Cancer Ther..

[47] Sehdev V., Katsha A., Ecsedy J., Zaika A., Belkhiri A., El-Rifai W. (2013). The combination of alisertib, an investigational aurora kinase A inhibitor, and docetaxel promotes cell death and reduces tumor growth in preclinical cell models of upper gastrointestinal adenocarcinomas. Cancer.

[48] Sehdev V., Peng D., Soutto M., Washington M.K., Revetta F., Ecsedy J., Zaika A., Rau T.T., Schneider-Stock R., Belkhiri A. (2012). The aurora kinase A inhibitor MLN8237 enhances cisplatin-induced cell death in esophageal adenocarcinoma cells. Mol. Cancer Ther..

[49] Yusenko M.V., Biyanee A., Frank D., Köhler L.H.F., Andersson M.K., Khandanpour C., Schobert R., Stenman G., Biersack B., Klempnauer K.-H. (2022). Bcr-TMP, a novel nanomolar-active compound that exhibits both MYB- and microtubule-inhibitory activity. Cancers.

[50] Köhler L.H.F., Reich S., Yusenko M., Klempnauer K.-H., Shaikh A.H., Ahmed K., Begemann G., Schobert R., Biersack B. (2022). A new naphthopyran derivative combines c-Myb inhibition, microtubule-targeting effects, and antiangiogenic properties. ACS Med. Chem. Lett..

[51] Köhler L.H.F., Reich S., Yusenko M., Klempnauer K.-H., Begemann G., Schobert R., Biersack B. (2023). Multimodal 4-arylchromene derivatives with microtubule-destabilizing, anti-angiogenic, and MYB-inhibitory activities. Cancer Drug Resist..

[52] Brabender J., Lord R.V., Danenberg K.D., Metzger R., Schneider P.M., Park J.M., Salonga D., Groshen S., Tsao-Wei D.D., DeMeester T.R. (2001). Increased *c*-Myb mRNA expression in Barrett’s esophagus and Barrett’s-associated adenocarcinoma. J. Surg. Res..

[53] Peng D., Guo Y., Chen H., Zhao S., Washington K., Hu T., Shyr Y., El-Rifai W. (2017). Integrated molecular analysis reveals complex interactions between genomic and epigenomic alterations in esophageal adenocarcinomas. Sci. Rep..

[54] Zhang L., Wang X., Li Y., Han J., Gao X., Li S., Wang F. (2021). *c*-Myb facilitates immune escape of esophageal adenocarcinoma cells through the miR-145-5p/SPOP/PD-L1 axis. Clin. Transl. Med..

[55] Sheldrick G.M. (2015). SHELXT*—*Integrated space-group and crystal-structure determination. Acta Crystallogr. A.

[56] Sheldrick G.M. (2015). Crystal structure refinement with SHELX. Acta Crystallogr. C Struct. Chem..

[57] Farrugia L.J. (2012). WinGX and ORTEP for Windows: An update. J. Appl. Cryst..

[58] Macrae C.F., Sovago I., Cottrell S.J., Galek P.T.A., McCabe P., Pidcock E., Platings M., Shields G.P., Stevens J.S., Towler M. (2020). Mercury 4.0: From visualization to analysis, design and prediction. J. Appl. Cryst..

[59] Landegren U. (1984). Measurement of cell numbers by means of the endogenous enzyme hexosaminidase. Applications to detection of lymphokines and cell surface antigens. J. Immunol. Methods.

[60] Dandawate P., Subramaniam D., Panovich P., Standing D., Krishnamachary B., Kaushik G., Thomas S.M., Dhar A., Weir S.J., Jensen R.A. (2020). Cucurbitacin B and I inhibits colon cancer growth by targeting the Notch signaling pathway. Sci. Rep..

